# Phosphoproteomic Analysis of FLCN Inactivation Highlights Differential Kinase Pathways and Regulatory TFEB Phosphoserines

**DOI:** 10.1016/j.mcpro.2022.100263

**Published:** 2022-07-19

**Authors:** Iris E. Glykofridis, Alex A. Henneman, Jesper A. Balk, Richard Goeij-de Haas, Denise Westland, Sander R. Piersma, Jaco C. Knol, Thang V. Pham, Michiel Boekhout, Fried J.T. Zwartkruis, Rob M.F. Wolthuis, Connie R. Jimenez

**Affiliations:** 1 Amsterdam UMC, location VUmc, Vrije Universiteit Amsterdam, Human Genetics, Cancer Center Amsterdam, Amsterdam, The Netherlands; 2 Amsterdam UMC, location VUmc, Vrije Universiteit Amsterdam, Medical Oncology, Cancer Center Amsterdam, Amsterdam, The Netherlands; 3 University Medical Center Utrecht, Center for Molecular Medicine, Molecular Cancer Research, Utrecht, The Netherlands; 4 Oncode Institute, Amsterdam, The Netherlands

**Keywords:** ACN, acetonitrile, BHD, Birt–Hogg–Dubé, DMEM, Dulbecco's modified Eagle's medium, FLCN, folliculin, IP, immunoprecipitation, INKA, integrative inferred kinase activity, PTM-SEA, posttranslational modification signature enrichment analysis, MS/MS, tandem mass spectrometry, pY, phosphotyrosine, pSTY, phosphoserine/threonine/tyrosine, RPTEC, renal proximal tubular epithelial cell, RCC, renal cell carcinoma, ROS, reactive oxygen species, RTK, receptor tyrosine kinase, TFEB, transcription factor EB

## Abstract

In Birt–Hogg–Dubé (BHD) syndrome, germline loss-of-function mutations in the Folliculin (*FLCN*) gene lead to an increased risk of renal cancer. To address how FLCN inactivation affects cellular kinase signaling pathways, we analyzed comprehensive phosphoproteomic profiles of FLCN^POS^ and FLCN^NEG^ human renal tubular epithelial cells (RPTEC/TERT1). In total, 15,744 phosphorylated peptides were identified from 4329 phosphorylated proteins. INKA analysis revealed that FLCN loss alters the activity of numerous kinases, including tyrosine kinases EGFR, MET, and the Ephrin receptor subfamily (EPHA2 and EPHB1), as well their downstream targets MAPK1/3. Validation experiments in the BHD renal tumor cell line UOK257 confirmed that FLCN loss contributes to enhanced MAPK1/3 and downstream RPS6K1/3 signaling. The clinically available MAPK inhibitor Ulixertinib showed enhanced toxicity in FLCN^NEG^ cells. Interestingly, FLCN inactivation induced the phosphorylation of PIK3CD (Tyr524) without altering the phosphorylation of canonical Akt1/Akt2/mTOR/EIF4EBP1 phosphosites. Also, we identified that FLCN inactivation resulted in dephosphorylation of TFEB Ser109, Ser114, and Ser122, which may be linked to increased oxidative stress levels in FLCN^NEG^ cells. Together, our study highlights differential phosphorylation of specific kinases and substrates in FLCN^NEG^ renal cells. This provides insight into BHD-associated renal tumorigenesis and may point to several novel candidates for targeted therapies.

## Highlights

• Phosphoproteomic profiles of FLCN^POS^ and FLCN^NEG^ human renal epithelial cells.
• Dependency on MAPK1/3 signaling in FLCN^NEG^ renal (tumor) cells.
• FLCN loss induces ROS and decreases TFEB Ser109, Ser114, and Ser122 phosphorylation.
• FLCN-directed kinase pathways may offer novel insights in BHD tumorigenesis.

## Graphical Abstract

**Figure:**
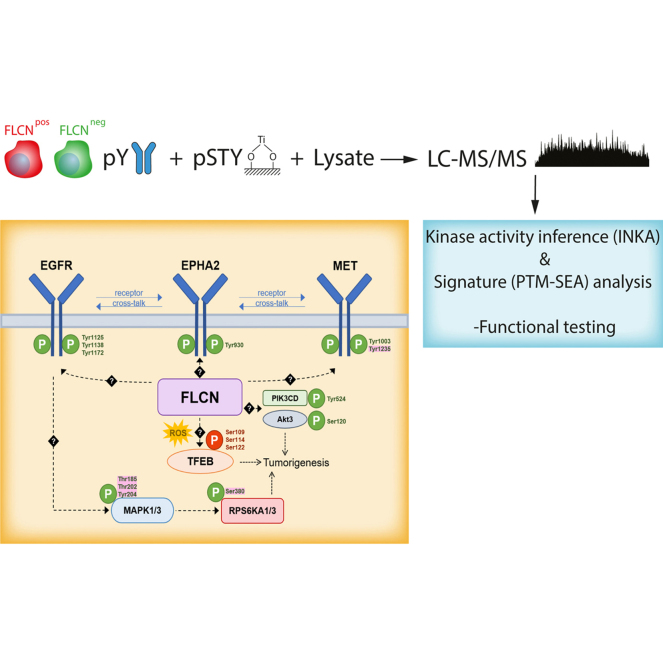

## In Brief

Comprehensive phosphoproteomics (pY + pSTY) of FLCN^POS^ and FLCN^NEG^ human renal cells in conjunction with INKA and with posttranslational modification–based signature enrichment analyses identified FLCN phosphorylation dependencies. FLCN-dependent kinase pathways were investigated through drug experiments and validated in a BHD tumor cell line. This pinpointed RTK-MAPK1/3-RPS6K1/3 as a key axis downstream of FLCN loss. In addition, we show that FLCN loss induces ROS and modulates localization of the TFEB transcription factor by dephosphorylation of specific serines.

In Birt–Hogg–Dubé (BHD) syndrome, germline mutations in the Folliculin (*FLCN*) gene predisposes carriers to an increased risk of renal cancer (1, 2). Loss of heterozygosity, by gene silencing or an inactivating somatic mutation of the wildtype *FLCN* allele, precedes the development of bilateral and multifocal renal tumors in patients BHD (3, 4, 5). Currently, there is no difference in the treatment of hereditary and sporadic renal cell carcinoma (RCC). Standard treatments include radio- and chemotherapy, partial or radical nephrectomy, and systemic (immuno)therapy (6). BHD carriers are dependent on life-long surveillance by renal imaging for early detection and treatment of RCC (7).

To gain more insight into the pathways by which FLCN suppresses renal tumorigenesis, we created FLCN^POS^ and FLCN^NEG^ human renal tubular epithelial cell lines to model BHD syndrome *in vitro*. Recently, we revealed FLCN-dependent gene and protein expression changes and identified that loss of FLCN induces two distinct transcriptional programs in renal epithelial cells. The first is characterized by expression of E-box controlled genes through activation of the basic helix–loop–helix leucine zipper transcription factors TFE3/TFEB, resulting in a specific autophagy and lysosomal gene expression signature in FLCN-deficient cells. The second program induces a set of genes under the control of interferon-stimulated response elements by means of STAT1/2 activation, which appears to counterbalance TFE-directed hyperproliferation (8).

Here, we investigate how FLCN influences cellular signaling pathways *via* protein and receptor phosphorylation by determining the comprehensive phosphoproteomic profiles and associated signaling pathways of FLCN^POS^ and FLCN^NEG^ human renal tubular epithelial cell lines. We pinpoint distinct regulatory phosphorylation events induced by FLCN loss that may link to early steps in the oncogenic transformation of renal cells. These insights warrant further investigation into renal tumorigenesis in patients with this hereditary cancer predisposition to expand current treatment options.

## Experimental procedures

### Cell Culture

Renal proximal tubular epithelial cells (RPTEC/TERT1, ATCC CRL-4031) were maintained in Dulbecco's modified Eagle's medium (DMEM)/F12 (Gibco, Life Technologies) according to ATCC’s protocol with addition of 2% fetal bovine serum (Gibco, Life Technologies). To maintain the selective pressure for immortalization, 0.1 mg/ml G418 Sulfate (Calbiochem) was added.

BHD renal tumor cell line UOK257 and its FLCN-reconstituted version UOK257-2 (9, 10) were kindly provided by Laura Schmidt and maintained in DMEM (Gibco, Life Technologies) with 8% fetal bovine serum (Gibco, Life Technologies). To maintain the selective pressure for FLCN expression in UOK257-2, the medium was supplemented with 2 μg/ml Blasticidin (Invitrogen, Life Technologies).

Cell lines were cultured in a humidified atmosphere at 37 °C and 5% CO2 and were regularly tested for mycoplasma contamination.

### Gene Editing

CRISPR/Cas9-mediated generation of FLCN knockout RPTEC cell lines was described earlier (8). In short, an inducible Cas9 RPTEC cell line was lentivirally created using the Lenti-X Tet-On 3G Inducible Expression System (Clontech, Takara Bio) and pLVX-Tre3G and Tre3G-Cas9 plasmids. To improve targeting efficiency, we simultaneously knocked out TP53 and FLCN, as it is known that a TP53-dependent DNA damage response inhibits effective gene editing in some cell types (11, 12). To disrupt the *TP53* and/or *FLCN* gene, synthetic gRNAs targeting 5′ exons were cotransfected, and Nutlin-3 (10 μM, Selleck Chemicals) was added for the selection of TP53 knockout and thus successfully transfected cells.

The following crRNA sequences were used: *FLCN*_exon 5 (GTGGCTGACGTATTTAATGG) *FLCN*_exon 7 (TGTCAGCGATGTCAGCGAGC), *TP53*_exon 4 (CCATTGTTCAATATCGTCCG). RPTEC hTERT (“WT”), RPTEC tet-on Cas9 (“Cas9”), and RPTEC tet-on Cas9 TP53^-/-^ (“TP53KO”) were FLCN wildtype and assigned to the FLCN^POS^ group. Three individually isolated RPTEC tet-on Cas9 TP53^-/-^ FLCN^-/-^ clones (“FLCNKO_C1,” “FLCNKO_C2,” and “FLCNKO_C3”) were assigned to the FLCN^NEG^ group. C1 and C2 were created with gRNAs targeting *FLCN* exon 5, and C3 was created with a gRNA targeting *FLCN* exon 7. The FLCN^KO^ RPTEC cell line used for validation experiments was created using Synthego’s Synthetic cr:tracrRNA Kit according to the protocol of the manufacturer. Cas9/gRNA (*FLCN*_exon 4 GAGAGCCACGAUGGCAUUCA + modified EZ scaffold) RNP complexes were transiently transfected using Neon Electroporation System (ThermoFisher). Subsequently, transfected cells were grown in limiting dilution in 96-well plates to generate single cell clones. The FLCN knockout status of clonal cell lines was verified by Sanger sequencing and Western blot (supplemental Fig. S1*A*).

### Sample Preparation and Phosphopeptide Enrichment

Sample preparation and immunoprecipitation (IP) procedures were performed as reported (13, 14, 15). In short, cell lines were harvested at 70 to 80% confluency (when cells are still growing exponentially) and lysed in urea lysis buffer (8 M urea, 1 mM orthovanadate, 2.5 mM pyrophosphate, 1 mM β-glycerophosphate in MilliQ water) followed by 1 min of vortexing and sonication. Next, lysates were cleared by centrifugation at 5400*g* for 15 min at 13 °C. The protein content was determined using the DCTM Protein Assay (Bio-Rad), and sample quality was examined by SDS-PAGE and Coomassie Blue staining (supplemental Fig. S1*B*). Global tyrosine phosphorylation levels among the samples were assessed by Western blot (1:1500, p-Tyr-1000 antibody, Cell Signaling Technology) (supplemental Fig. S1*C*).

For each cell line, dithiothreitol (DTT) (4 mM, 30 min at 55 °C) was added to 5 mg of protein, followed by iodoacetamide (10 mM, 15 min in the dark). The solution was then diluted to 2 M Urea by the addition of 20 mM Hepes pH 8.0 and digested with trypsin (Promega) at a final concentration of 5 μg/ml overnight (room temperature). The digests were then acidified with trifluoroacetic acid (TFA) to a final concentration of 0.1% and desalted using (500 mg) Oasis HLB columns (Waters). Columns were equilibrated in 0.1% TFA. Subsequently, bound peptides were washed twice with 0.1% TFA, eluted in 0.1% TFA/80% acetonitrile (ACN) and lyophilized. pTyr IP was performed using PTMScan pTyr antibody beads (p-Tyr-1000 antibody, Cell Signaling Technology) at a ratio of 4 μl bead slurry per mg protein. Eluted phosphopeptides were desalted using a STAGE tip containing SDB-XC material (3M). Lysate aliquots were taken before the pTyr IP step and diluted to 0.1 μg/μl in 0.1% TFA for proteomic analysis.

Subsequently, Titanium dioxide (TiOx) chromatography was applied to capture the remaining phosphopeptides. Desalted tryptic digests, 500 μg, were diluted 1:1 with lactic acid solution (0.3 g/ml lactic acid, 0.07% TFA/53% acetonitrile). Pipette tips (200 μl) were fitted with a 16G-needle punch of a C8 disk EMPORE, on which 2.5 mg TiO_2_ was added. The TiOx bed was preconditioned with 0.1% TFA and 80% acetonitrile before equilibration with 0.3 g/ml lactic acid in 0.07% TFA/54% acetonitrile, allowing the capture of phosphorylated serine and threonine peptides from the tryptic digest. After sequential washing of the bedding with lactic acid and 0.1% TFA +80% acetonitrile, the phosphopeptides were eluted with 0.5% and 5% (v/v) piperidine in 20% (v/v) phosphoric acid to quench the basic solution. Pipette tips (200 μl) were again fitted with a 16G-needle punch of an EMPORE disk of polyStyreneDivinylBenzene material, preconditioned with 0.1% TFA and 80% acetonitrile, and equilibrated with 0.1% TFA. After loading the enriched phosphopeptide mixture, the bedding was washed with 0.1% TFA. Through centrifugal filtration, the phosphopeptides were desalted in 0.1% TFA and 80% acetonitrile and lyophilized. The peptides were redissolved in loading solvent (0.5% TFA/4% acetonitrile) prior to separation on an Ultimate 3000 nanoLC-MS/MS system (Dionex LC-Packings) equipped with a 20 cm × 75 μm ID fused silica column. A volume of 18 μl was injected using partial loop injection.

For UOK257 and UOK257-2, samples were prepared using a slightly modified protocol, using a 9 M urea lysis buffer with 20 mM Hepes pH 8.0, 1 mM sodium orthovanadate, 2.5 mM sodium pyrophosphate, and 1 mM β-glycerophosphate in MilliQ water. Also, global phosphopeptide enrichment was performed from 200 μg peptides using IMAC cartridges on a BRAVO Assaymap liquid handler (Agilent) according to the protocol of the manufacturer. The IMAC elution solvent was 5% NH4OH in 30% ACN. UOK257 and UOK257-2 lysates (1 μg) were diluted from the desalted digest to 0.1 μg/μl, and 10 μl was injected for single-shot analysis.

### Phosphopeptide and Phosphosite Identification and Quantification

Peptides were separated by an Ultimate 3000 nanoLC-MS/MS system (ThermoFisher) equipped with a 50 cm × 75 μm ID Acclaim Pepmap (C18, 1.9 μm, ThermoFisher) column. After injection, peptides were trapped at 3 μl/min on a 10 mm × 75 μm ID Acclaim Pepmap trap at 2% buffer B (buffer A: 0.1% formic acid [Fischer Scientific], buffer B: 80% ACN, 0.1% formic acid) and separated at 300 nl/min in a 10 to 40% buffer B gradient in 90 min (125 min inject-to-inject) at 35 °C. Eluting peptides were ionized at a potential of +2 kVa into a Q Exactive HF mass spectrometer (ThermoFisher). Intact peptide masses were measured at resolution 120,000 in the Orbitrap using an AGC target value of 3E6 charges. The top 15 peptide signals (charge-states 2+ and higher) were submitted to tandem mass spectrometry (MS/MS) in the higher-energy collision cell (1.4 amu isolation width, 2526% normalized collision energy). MS/MS spectra were acquired at resolution 15,000 in the orbitrap using an AGC target value of 1E6 charges, a MaxIT of 64 ms, and an underfill ratio of 0.1%. Dynamic exclusion was applied with a repeat count of 1 and an exclusion time of 30 s.

MS/MS spectra were searched against the Swissprot human reference proteome FASTA file (2018_01, 42.258 entries) using MaxQuant 1.6.0.16. For UOK257, Swissprot human reference proteome FASTA file (2021_01, 42,383 entries) and MaxQuant 1.6.10.43 were used. Enzyme specificity was set to trypsin and up to two missed cleavages were allowed. Cysteine carboxamidomethylation (Cys, +57.021464 Da) was treated as fixed modification and serine, threonine, and tyrosine phosphorylation (+79.966330 Da); methionine oxidation (Met,+15.994915 Da); and N-terminal acetylation (N-terminal, +42.010565 Da) as variable modifications. Peptide precursor ions were searched with a maximum mass deviation of 4.5 ppm and fragment ions with a maximum mass deviation of 20 ppm. Peptide, protein, and site identifications were filtered at a false discovery rate of 1% using the decoy database strategy. The minimal peptide length was seven amino acids, the minimum Andromeda score for modified peptides was 40, and the corresponding minimum delta score was 6 (default MaxQuant settings). Peptide identifications were propagated across samples using the match between runs option checked. Lysate searches were performed with the label-free quantification option selected. Phosphosites were quantified by their extracted ion intensities (“Intensity” in MaxQuant). For downstream analysis only class I phosphosites with localization probability >0.75 were used. For each pTyr IP sample the phosphosite intensities were normalized (“normalized intensity”) on summed total intensity of the corresponding lysate. For global phosphoproteomics data using TiO_2_, phosphosite intensities were log-transformed and median normalized. Statistical analysis of phosphosite data was performed using limma (16) with the site multiplicity retained. Missing values were imputed. Data are available *via* ProteomeXchange with identifier PXD025798 (RPTEC) and PXD030237 (UOK257).

### Statistics and Data Analyses

MaxQuant protein database searches for both pTyr immunoprecipitated (pY) and global phosphoenriched datasets (pSTY) were performed separately as described in the previous section, and their results were subsequently exported into TXT files. From each set of exported results, the files modificationSpecificPeptides.txt, Phospho (STY)Sites.txt, evidence.txt, and experimentaDesignTemplate.txt were used as inputs for two separate integrative inferred kinase activity (INKA) analyses (16). Code for this analysis was downloaded from https://inkascore.org/ and run from the Linux command-line. After running the script read_MQ_tables.R, the script inka.R was run using the flags –dump.substrate –no.network. Subsequently, the provided script collect_output_tables.R was used to compose tsv-format tables of all kinase inkascores and also activation-loop scores. The pY and pSTY inkascore tables and in-house R scripts were used for the generation of all INKA kinase activity boxplots using default R functions. The kinase inkascore horizontal barplots are extracted from the standard output of the inka.R script.

For the construction of the hybrid pY+pSTY networks, in-house scripts were used to combine inka_table.tsv, actloop_table.tsv, PSP_substrates.txt, and NWK_substrates.txt tables from both pY and pSTY datasets as a direct sum. The resulting hybrid data were subsequently plotted using the same network plotting function, generate_KSR_network, defined in the inka.R script.

The posttranslational modification–based gene set enrichment analysis (PTM-SEA) was based solely on the MaxQuant search result export file Phospho (STY)Sites.txt and performed on pY and pSTY datasets separately. All sites belonging to either decoy or contaminant sequences were removed, and only phosphosites with a localization probability ≥0.75 (class I) were retained. Next, only the “__1” (single phosphorylation) site intensities in each sample were used. Missing values were imputed by a value of 1, and all intensities were log10 transformed. The resulting data matrices were submitted to a two-group comparison using limma version 3.46.0. The resulting *p*-value (p.value) and fold changes (FC) were used to generate a rank value rank.value = 10∗sign(FC)∗log10(p.value) for each site. The resulting matrix was saved into gct-format file using an in-house developed R script. The gct format input file was used as input for the PTM-SEA described in (17). This analysis was performed on a local workstation using the script ssGSEA2.0.R available from https://github.com/broadinstitute/ssGSEA2.0, using the set definition file ptm.sig.db.all.flanking.human.v1.9.0.gmt obtained from http://prot-shiny-vm.broadinstitute.org:3838/ptmsigdb-app/. The resulting output files were further processed using in-house developed R scripts to produce the provided set enrichment bar plots.

### Drug Sensitivity Assays

For generation of dose–response curves RPTEC cells were seeded in 100 μl medium in 96-well plates (2000 cells/well). For BHD tumor cell line UOK257 a seeding density of 1500 cells/well was used. On the next day, receptor tyrosine kinase (RTK) inhibitors (Crizotinib [S1068], Foretinib [S1111], Silmitasertib [S2248], Erlotinib [S7786], and Ulixertinib [S7854], Selleckchem) were added using the Tecan D300e Digital Dispenser and corresponding software. After a 72 h treatment, cell viability was assessed by a 6 h incubation with 20 μl of CellTiter-Blue reagent (Promega) at 37 °C. Fluorescence (560Ex/590Em) was measured in a microplate reader (TriStar LB 941, Berthold Technologies). All experiments were performed at least in triplicate, with three technical replicates within each experiment. Cell line–specific dose–response curves were created in GraphPad Prism. Statistically significant differences between FLCN^POS^ and FLCN^NEG^ were identified by fitting ANOVA-like models (using cell line and dose as covariates) assuming the data were beta distributed, *i.e.*, all viability frequencies were between 0 and 100%.

### Amino Acid Starvations and Immunofluorescence

For immunofluorescence experiments, cells were grown on coverslips (15 mm diameter). After 24 h, the medium was refreshed or replaced by serum-free medium. Amino acid starvation was done with cells that had been serum starved overnight in custom-made DMEM without amino acids but containing 2 mM L-glutamine for 2 h. Next, cells were washed twice with ice-cold PBS and fixed in 4% para-formaldehyde for 20 min at room temperature. After washing with PBS, cells were permeabilized with 0.25% Saponin (Sigma Aldrich) in PBS and blocked with blocking solution (1% FCS, 0.25% Saponin in PBS). For cell lines overexpressing TFEB 0.1% Triton-X100 was used for permeabilization. Coverslips were incubated overnight at room temperature with primary antibodies in blocking solution. On the next day, coverslips were washed in 0.25% Saponin in PBS and incubated with secondary antibodies from Fisher Scientific (Alexa 488-goat anti-mouse; 10696113 and Alexa 568-goat anti-rabbit; A11036) for 2 h at room temperature. Cells were then mounted with Immu-Mount (Thermo Scientific Shandon). Specimens were visualized under Zeiss LSM510 confocal or Zeiss AxioObserver Z1 inverted microscopes and imaged using Zeiss vision software. The following antibodies were used: TFEB (CST 3778, D2O7D; 1:100), FLAG (for TFEB overexpression, F3165; Sigma; 1:100), Lamp2 (H4B4, Ab24631; Abcam; 1:400), and TFE3 (CST 14779; 1:300). For Western blot analyses of starvation experiments, cells were seeded in 6-well plates and treated as outlined above. Instead of fixation, cells were scraped into 1× Laemmli sample buffer (Sigma Aldrich) and immunoblotted as described below. Starvations, as well as immunofluorescent stainings and immunoblotting, were performed twice. To check the specificity of the antibody, a siTFEB (ON-TARGETplus Human TFEB siRNA, L-009798-00-0005, Dharmacon, Horizon) treated condition was also performed (supplemental Fig. S7, *D* and *E*).

### Immunoblotting

For immunoblotting cell pellets were lysed in RIPA Lysis and Extraction Buffer (89900, ThermoFisher Scientific) supplemented with phosphatase and protease inhibitors (Roche). Subsequently, samples were boiled at 70 °C for 5 min in 1× NuPAGE LDS sample buffer (Novex NP0007, ThermoFisher) with 10% 1 M DTT (Sigma), and equal amounts were separated by 4 to 15% or 10% SDS-PAGE (Bio-Rad). Proteins were transferred onto polyvinylidene fluoride (PVDF) membranes (Merck), and subsequently blocked for 1 h at room temperature with 5% milk (ELK, Campina) in TBST. The primary antibody incubation was overnight at 4 °C in 2.5% milk in TBST. On the next day, the membrane was washed and incubated with the appropriate secondary antibodies (Dako) for 3 h at 4 °C in 2.5% milk in TBST. For detection of phosphorylated proteins, blocking and incubation steps were performed with Bovine Serum Albumin (BSA Fraction V, Roche) instead of milk. After incubation the membrane was thoroughly washed and bands were visualized by chemoluminescence (ECL prime, Amersham, VWR) in combination with ChemiDoc Imaging Systems (Bio-Rad).

### Image Analysis

For quantifications of nuclear and cytoplasmic TFEB immunofluorescent intensities, images were segmented using the ZeroCostDL4Mic platform (17), running Cellpose (18) with the “Cyto 2” model with default settings to detect the outer border of cytoplasm of cells. Segmented images were used as input for a script to semiautomatically determine size of individual cells and nuclei. All images were visually inspected, and cells on the border of the image, multinucleated or missegmented cells were discarded. Subsequently, ratio values were calculated as mean intensity of nuclear TFEB divided by the mean intensity of cytoplasmic TFEB. ImageJ script is available: https://github.com/Boekhout/imageJ_TFEB

### Antibodies

For Western blot experiments the following antibodies were used: FLCN (D14G9, CST 3697S, 1:1000), RRAGD (CST 4470S, 1:1000), TFE3 (CST 14779, 1:1000), TFEB (CST 3778, D2O7D; 1:3000), GAPDH (sc-47724 and MAB374; Merck Millipore; 1:5000), and Tubulin (D-10: sc-5274; 1:5000). Antibodies and concentrations used for immunofluorescence are described in the “Amino acid starvations and immunofluorescence” section above.

### Virus Production and Infection

To create RPTEC cell lines that overexpress TFEB wildtype (WT) or TFEB phosphomutants (TFEB Ser109, Ser114, Ser122 S>A and S>D), PCR fragments were derived from TFEB expression constructs kindly gifted by Rosa Puertollano (19). These were subcloned into pLenti CMVie-IRES-BlastR (a gift from Ghassan Mouneimne, Addgene plasmid #119863 (20)) and verified by plasmid sequencing. Next, lentiviral particles were produced in HEK293T cells and transduced into RPTEC tet on Cas9 TP53 KO (FLCN^POS^) and RPTEC tet on Cas9 TP53 KO FLCN KO C3 (FLCN^NEG^) cells. Blasticidin (15 μg/ml, Invitrogen, Life Technologies) was added for selection of successfully transduced cells and protein overexpression was confirmed by Western blotting (Fig. 7*A*).

### Oxidative Stress Detection

To measure reactive oxygen species (ROS) levels, we used the CellROX Green Reagent (Thermo Scientific) according to the manufacturer’s protocol. Menadione treatment (100 μM for 1 h at 37 °C) was used as a positive control. After labeling with CellROX Green Reagent, samples were collected by trypsinization, washed 1× in PBS (500*g*, 5 min), fixed in 4% paraformaldehyde (RT, 15 min) and stored at 4 °C. Samples were measured the subsequent day. ROS levels were quantified by recording green fluorescence (FITC/GFP detector) of at least 20,000 single cells per sample, on a BD LSR Fortessa Cell Analyzer flow cytometer (BD Biosciences), and the data were analyzed using FlowJo (V10.1.1).

### Experimental Design and Statistical Rationale

Phosphoproteomic profiling was performed using reproducible label-free workflows (13, 21). To this end, phosphotyrosine-based immune precipitation and titanium oxide–based global phosphopeptide capture was applied to six different RPTEC/TERT1 cell lines with different genetic profiles created by CRISPR/Cas9-mediated gene editing. Three cell lines express wildtype FLCN (grouped as “FLCN^POS^”) and three cell lines are FLCN knockout (groups as “FLCN^NEG^”). Each FLCN knockout cell line harbors a unique FLCN mutation that caused protein disruption. The maximum number of replicates per condition was based on effectively handling samples without compromising the quality of processing. For UOK257 and UOK257-2, lysates were harvested in duplicate and phosphopeptides were captured using phosphotyrosine-based immune precipitation and IMAC. To avoid batch effects in mass spectrometry measurements, samples and conditions were alternated.

The R package limma (16) was used to perform differential phosphorylation analysis of ion intensity data, because it was designed for handling of differential expression studies and smaller sample sizes. For each pTyr IP sample, the phosphosite intensities were normalized (“normalized intensity”) on summed total intensity of the corresponding lysate. For global phosphoproteomics data using TiO2, phosphosite intensities were log-transformed and median normalized. Statistical aspects of INKA scoring and PTM-SEA have been described in the original publications (22).

For drug sensitivity assays, cell lines were tested for multiple inhibitors at least in triplicate, with three technical replicates in each experiment. Each drug response curve shows relative viability with the standard deviation indicated. Statistically significant differences between FLCN^POS^ and FLCN^NEG^ were identified by fitting ANOVA-like models (using cell-line and dose as covariates) assuming the data were beta distributed, *i.e.*, all viability frequencies were between 0 and 100%.

## Results

### FLCN-Positive and FLCN-Negative Renal Epithelial Cells Show Distinct Phosphorylation Patterns

To investigate the effects of kidney-specific FLCN loss on kinase hyperactivity and intracellular protein phosphorylation, we used FLCN^POS^ and FLCN^NEG^ renal proximal tubular epithelial cell models (RPTEC/TERT1) (8). In short, expression of the *FLCN* gene was disrupted using CRISPR/Cas9-mediated gene editing and three independent FLCN knockout cell lines were created. As TP53-dependent DNA damage response inhibits effective gene editing in some cell types (11, 12), we simultaneously knocked out TP53 and FLCN. To study FLCN-specific effects, we used three separate FLCN^POS^ cell lines for comparison, as described in a previous study (8).

**Fig. 1:**
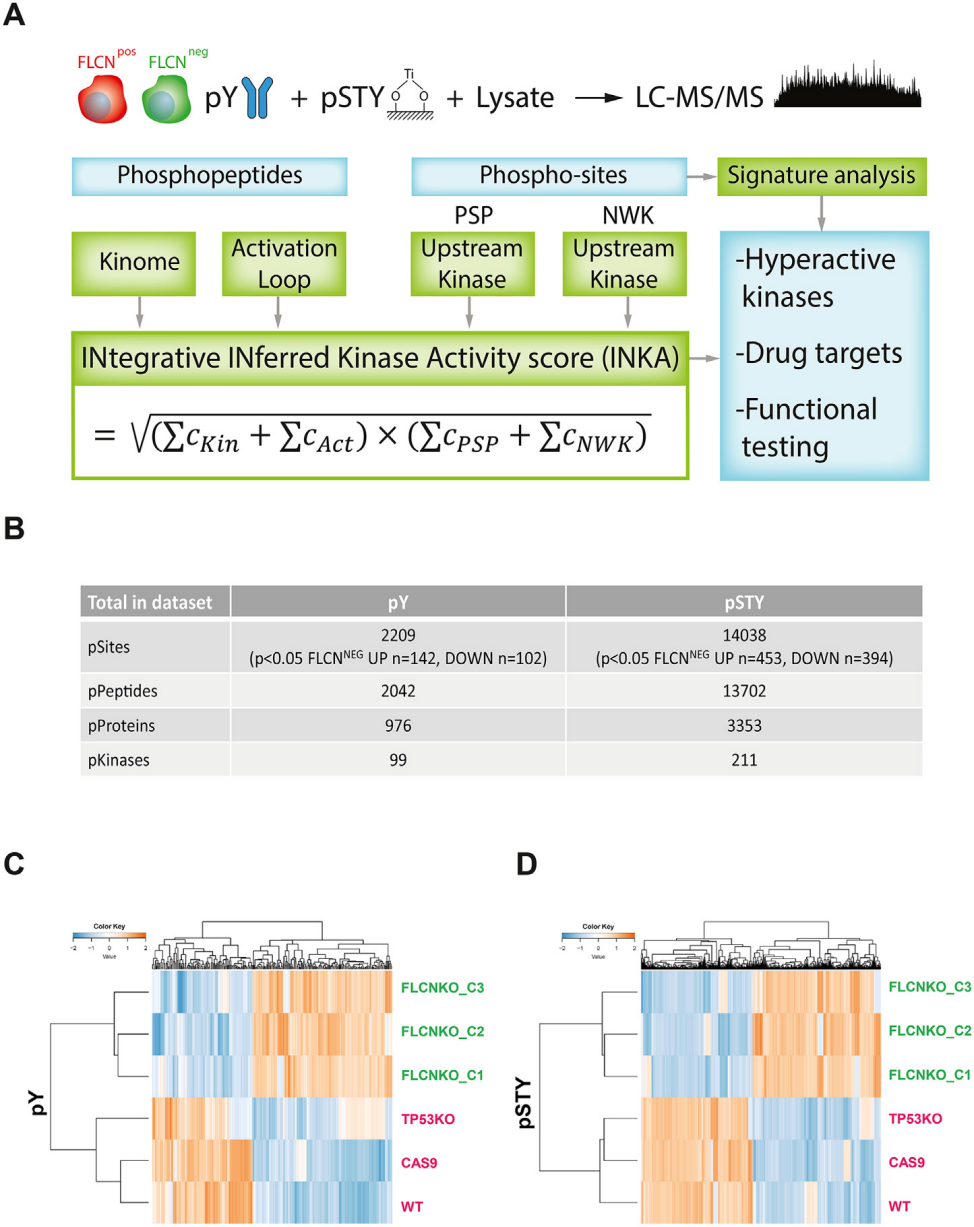
**Phosphoproteomic analyses of FLCN**^**POS**^**and FLCN**^**NEG**^**renal epithelial cells**. *A*, workflow of phosphoproteomic analyses of FLCN^POS^*versus* FLCN^NEG^ renal epithelial cells. To gain insight into FLCN-dependent activation of specific kinases we used the INKA algorithm (15), which takes into account both phosphorylation of the kinase itself (“kinome” and “activation loop”) and phosphorylation of substrate-specific sites (PhosphoSitePlus and NetworKIN; “PSP” and “NWK,” respectively). *B*, numbers of identified phosphorylated sites, peptides, proteins, and kinases in both datasets. Differential phosphosites between FLCN^POS^ and FLCN^NEG^ are indicated below the total number of identified phosphosites. *C*, supervised hierarchical cluster analyses of FLCN differential pY peptide intensities. RPTEC hTERT (WT), RPTEC tet-on Cas9 (Cas9), and RPTEC tet-on Cas9 TP53-/- (TP53KO) were FLCN wildtype and assigned to the FLCN^POS^ group. Three individually isolated RPTEC tet-on Cas9 TP53-/- FLCN-/- clones (FLCNKO_C1, FLCNKO_C2, and FLCNKO_C3) were assigned to the FLCN^NEG^ group. *D*, supervised hierarchical cluster analyses of FLCN differential pSTY peptide intensities. RPTEC hTERT (WT), RPTEC tet-on Cas9 (Cas9), and RPTEC tet-on Cas9 TP53-/- (TP53KO) were FLCN wildtype and assigned to the FLCN^POS^ group. Three individually isolated RPTEC tet-on Cas9 TP53-/- FLCN-/- clones (FLCNKO_C1, FLCNKO_C2, and FLCNKO_C3) were assigned to the FLCN^NEG^ group. FLCN, folliculin; RPTEC, renal proximal tubular epithelial cell.

The experimental outline of this phosphoproteomic study is shown in Figure 1*A*. Sequential phosphopeptide enrichment from FLCN^POS^ and FLCN^NEG^ cell line lysates was performed using phosphotyrosine (pY) immunoprecipitation, followed by capture using titanium dioxide (pSTY) on the flow-through fraction (supplemental Fig. S1, *A* and *B*). Subsequently, phosphoproteomic profiles were determined by label-free LC-MS/MS-based proteomics. An overview of mass spectrometry results is shown in Figure 1*B*. In total, the tyrosine-phosphorylation dataset consisted of 2042 peptides, with 976 phosphorylated proteins carrying 2209 phosphosites, of which 244 were significantly (*p* < 0.05) altered in FLCN^NEG^ cells (n = 142 up, n = 102 down). For the global phosphoproteome (mainly pSerine and pThreonine), 13,702 phosphorylated peptides were identified, with 3353 phosphorylated proteins and 14,038 phosphosites of which 847 were significantly (*p* < 0.05) altered in FLCN^NEG^ cells (n = 453 up, n = 394 down). Of the phosphorylated proteins, 310 are known kinases.

The bar graphs in supplemental Fig. S1, *D* and *E* show an overview of the number of identified phosphosites per individual cell line, with the fraction of singly, doubly, or triply phosphorylated sites indicated, showing the high consistency of the dataset. Hierarchical cluster analyses of FLCN differential phosphopeptide intensities revealed a clear separation between the two groups (Figs. 1, *C* and *D* and S1, *F* and *G*) revealing numerous phosphorylation events induced by FLCN loss.

For unsupervised clustering, all peptides were used, while for supervised clustering only the peptides that were differential between FLCN^POS^ and FLCN^NEG^ groups were used (taking into account class information and *p* < 0.05). Supplemental Fig. S2 shows a volcano plot of the pY FLCN^POS^ *versus* FLCN^NEG^ two-group comparison to convey the dataset-wide distribution of both *p*-values and measured amplitudes. Bar graphs of the top 20 show the most significantly changed pY phosphosites that are either more (supplemental Fig. S2*B*) or less (supplemental Fig. S2*C*) phosphorylated in FLCN^NEG^ RPTEC. For the pSTY FLCN^POS^ *versus* FLCN^NEG^ two-group comparison the volcano plot and the top 20 differential phosphosites is shown in supplemental Fig. S3. Both volcano plots show some discretization due to imputation of missing values. Taken together, we show that FLCN loss has a clear impact on the phosphoproteome in human renal epithelial cells.

### Kinase Activity Dependent on FLCN Expression

**Fig. 2:**
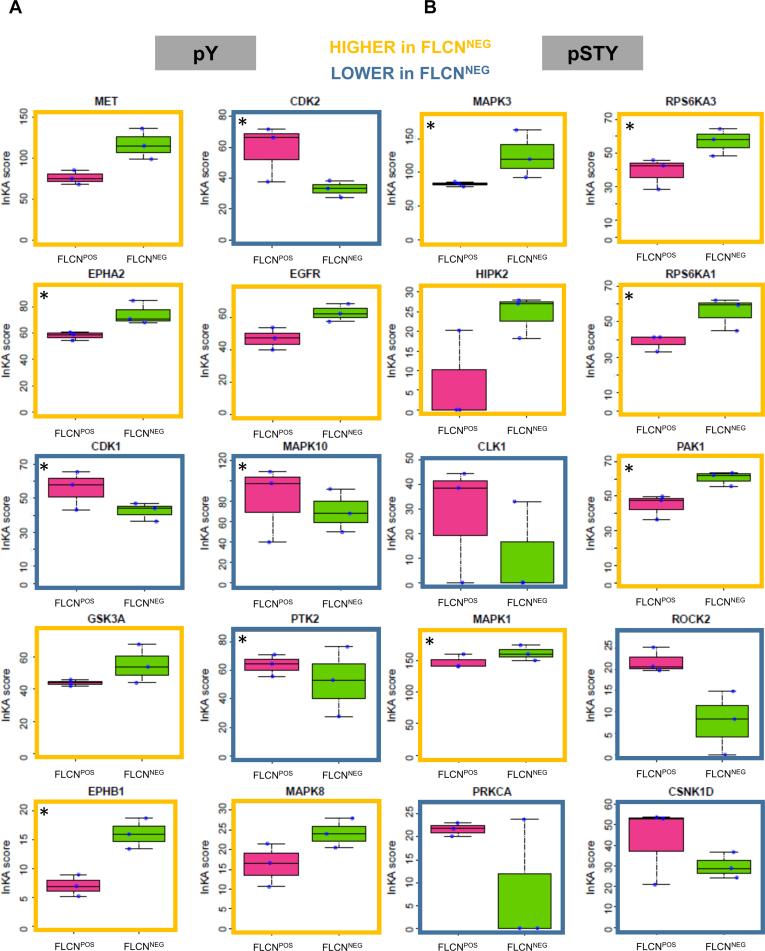
**INKA analyses of FLCN**^**POS**^***versus* FLCN**^**NEG**^**renal epithelial cells.***A*, *top* 10 differential kinases pY identified by INKA. Ranking is based on the difference of means of three observations per group. Kinases more active in FLCN^NEG^ cells are *boxed yellow*, and kinases more active in FLCN^POS^ are *boxed blue*. Kinase activity similarly dependent on FLCN in UOK257 cell line background are indicated by *asterisks*. Extended list of INKA results is attached as supplemental File S1. *B*, *top* 10 differential kinases pSTY identified by INKA. Ranking is based on the difference of means of three observations per group. Kinases more active in FLCN^NEG^ cells are *boxed yellow*, and kinases more active in FLCN^POS^ are *boxed blue*. Kinase activity similarly dependent on FLCN in UOK257 cell line background are indicated by asterisks. Extended list of INKA results is attached as supplemental File S1. FLCN, folliculin; INKA, integrative inferred kinase activity.

To gain insight into activation of specific kinases upon FLCN loss, we used the Integrative Inferred Kinase Activity (INKA) algorithm (22). As depicted in Figure 1*A*, the INKA algorithm calculates a score based on four components. It takes into account both phosphorylation of the kinase itself (“kinome” and “activation loop”) as well as phosphorylation of substrate specific sites (PhosphoSitePlus and NetworKIN; “PSP” and “NWK,” respectively). Based on this information INKA defines which kinases are most likely (hyper)activated in the specific sample measured. By default, INKA is used for the analyses of single samples but as we are interested in differentially phosphorylated kinase pathways dependent on FLCN expression, we grouped INKA scores of individual FLCN^POS^ *versus* FLCN^NEG^ RPTEC lines and calculated aggregated INKA scores to assess the universal effect of FLCN loss on kinase activation. Box plots of the top 10 differential kinases identified are shown in Figure 2, *A* (pY) and *B* (pSTY) and are based on the INKA score and ranked on difference of the means. A complete overview of INKA results is shown in supplemental Files S1 and S2. Kinases more active upon FLCN loss are outlined in yellow, whereas kinases less active are outlined in blue. We found more activity of tyrosine kinases EGFR, MET, and the Eph RTK subfamily (EPHA2 and EPHB1) upon FLCN loss. For threonine and serine phosphorylation, we detected more activity of MAPK1/3, ribosomal S6 kinases (*i.e.*, RPS6KA1 and RPS6KA3), and HIPK2 upon FLCN loss. Reduced kinase activity was observed for, among others, CDK1/2, MAPK10, ROCK2, and PRKCA.

The INKA-based rankings of top 20 active kinases identified per individual cell line are visualized as bar graphs in supplemental Fig. S4*A* (pY) and *B* (pSTY). Moreover, aggregated networks of kinases and substrates identified in either FLCN ^POS^ or FLCN^NEG^ cell lines, which were used to calculate aggregated INKA scores, are shown in supplemental Fig. S5. The aggregated INKA analysis identifies common kinase-substrate phosphorylation relations in FLCN^POS^ and FLCN^NEG^ cells, excluding individual cell line dependencies. This analysis shows that, in FLCN^NEG^ cells, EPHA2 is centrally located with many connections and activates MAPK1/3, while MET and EGFR have less connections and remain more peripherally located in the network. Further underscoring the potential importance of all differential kinase activities is the high EPHA2 rank in FLCN^NEG^ cell lines (rank 2/4/2 in FLCN^NEG^ C1/C2/C3, respectively), as compared with the FLCN^POS^ cell lines (rank 6/5/5 in FLCN^POS^ WT/Cas9/TP53^KO^, respectively) (supplemental Fig. S4). Similarly, EGFR ranks higher in FLCN^NEG^ cell lines (rank 5/3/6) *versus* FLCN^POS^ cell lines (rank 10/8/6). Finally, MET also ranks higher in FLCN^NEG^ cell lines (rank 1/1/1) *versus* FLCN^POS^ cell lines (rank 1/2/3). These INKA ranking results support the significant difference in kinase activities as shown by the box plots in Figure 2.

### Global Phosphosite-Specific Signaling Signatures Upon FLCN Inactivation

**Fig. 3:**
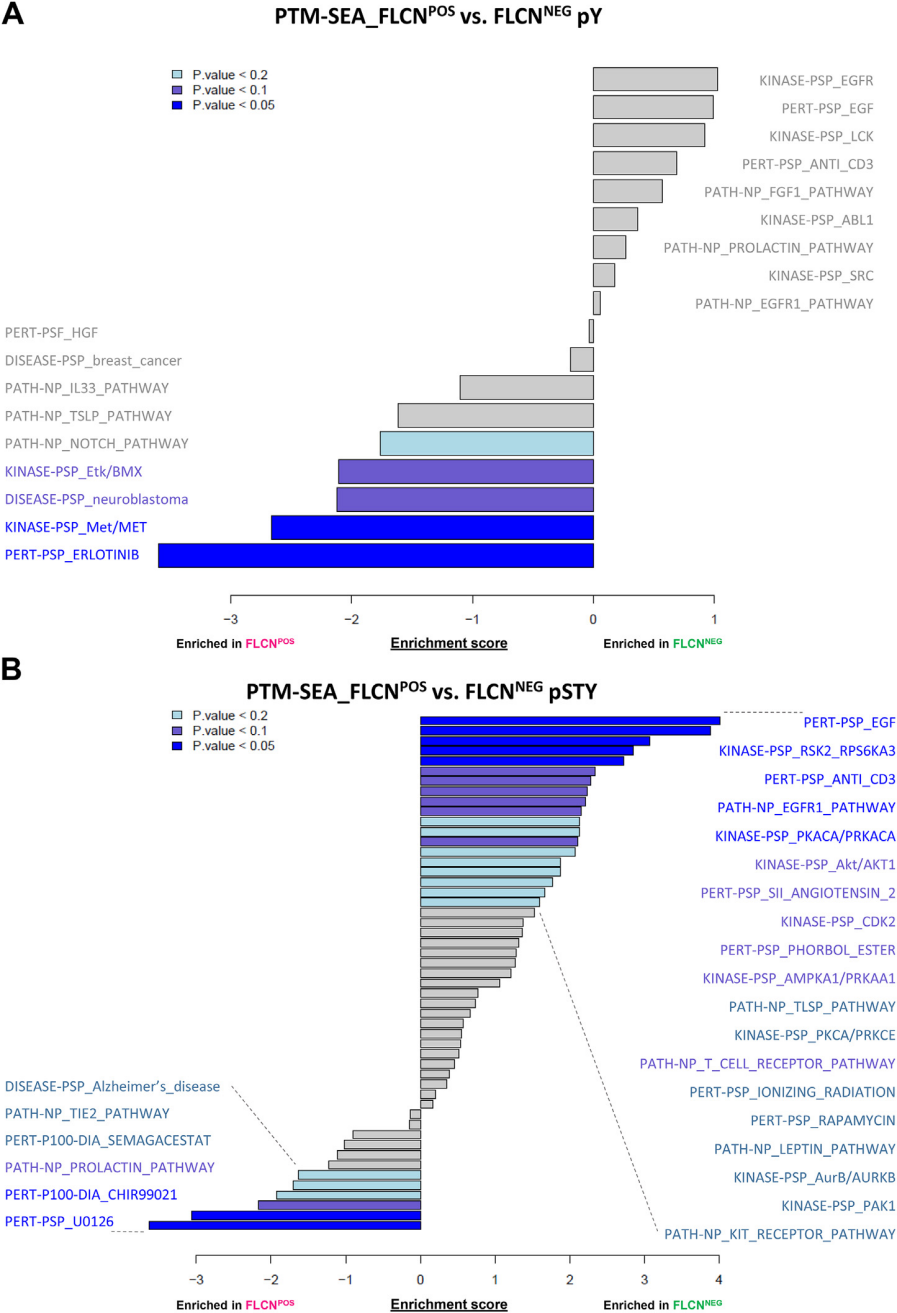
**Results of phosphosite-specific signature analyses (PTM-sigDB).***A*, results of phosphosite-specific signature analysis pY. Significance (*p*-value) of enriched signatures is indicated by *color*. *B*, results of phosphosite-specific signature analysis pSTY. Significance (*p*-value) of enriched signatures is indicated by *color*.

**Fig. 4:**
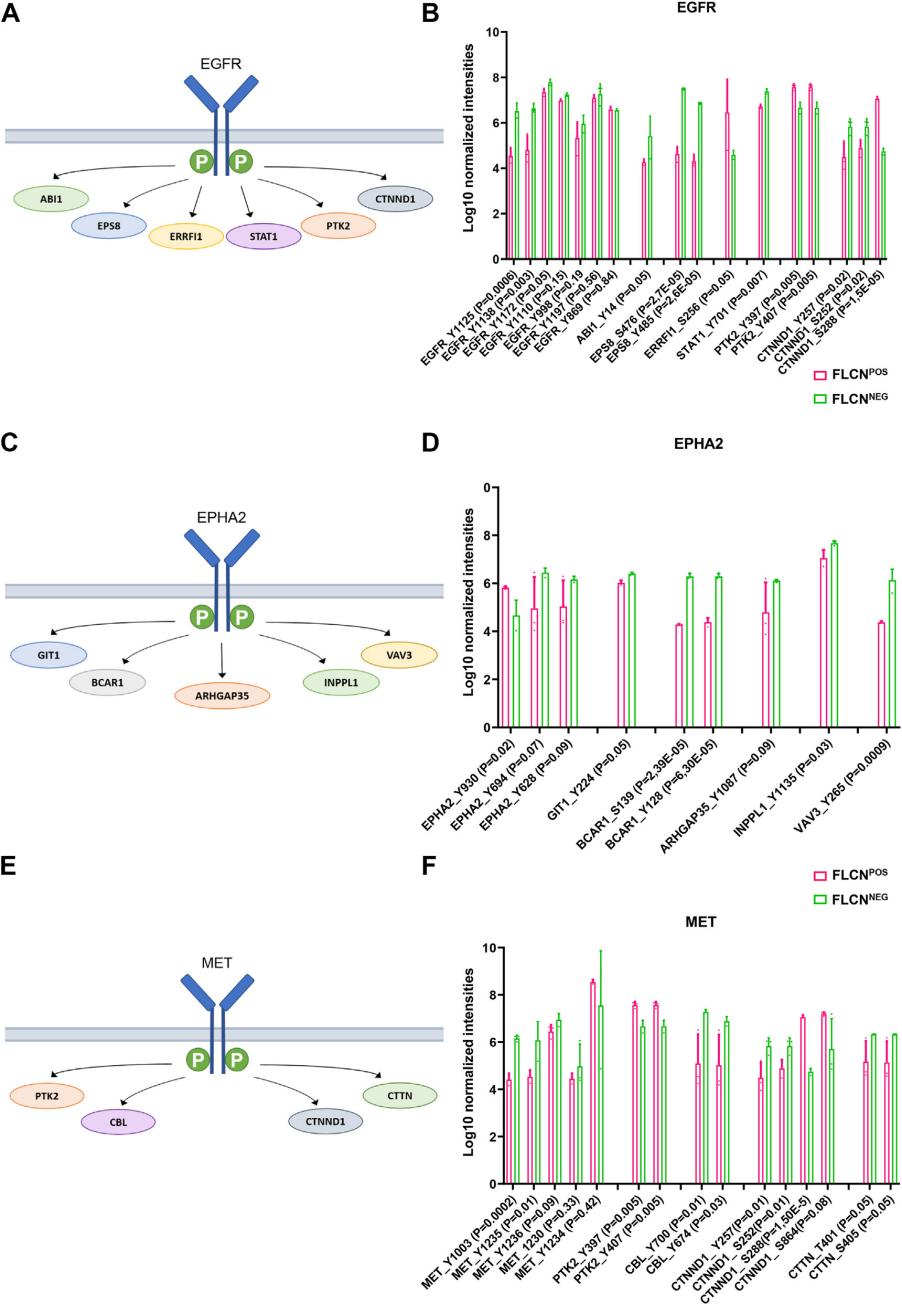
**Hyperphosphorylated tyrosine kinases in FLCN**^**NEG**^**renal epithelial cells.***A*, EGFR together with its substrates that were identified to be differentially phosphorylated upon FLCN loss in RPTECs. *B*, bar graph of normalized phosphosite intensities of EGFR and its substrates ABI1, EPS8, ERRFL1, STAT1, PTK2, and CTNND1. *p*-Values are indicated for each phosphosite depicted in the bar graph. *C*, EPHA2 together with its substrates that were identified to be differentially phosphorylated upon FLCN loss in RPTECs. *D*, bar graph of normalized phosphosite intensities of EPHA2 and its substrates GIT1, BCAR1, ARHGAP35, INPPL1, and VAV3. *p*-Values are indicated for each phosphosite depicted in the bar graph. *E*, MET together with its substrates that were identified to be differentially phosphorylated upon FLCN loss in RPTECs. *F*, bar graph of normalized phosphosite intensities of MET and its substrates PTK2, CBL, CTNND1, and CTTN. *p*-Values are indicated for each phosphosite depicted in the bar graph. FLCN, folliculin; RPTEC, renal proximal tubular epithelial cell.

To further assess signaling signatures dependent on FLCN loss, we performed phosphosite-specific signature analysis using PTM-SEA (23). We compared FLCN^POS^ *versus* FLCN^NEG^ RPTECs and present enriched pathways found in pY (Fig. 3*A*) and pSTY datasets (Fig. 3*B*) in bar graphs. When focusing on the most significant enriched gene sets (*p* < 0.05) this analysis reveals an enrichment of EGF and EGFR, PKACA/PRKACA, and RSK2/RPS6KA3 signaling, and an anti-CD3 signature in FLCN^NEG^ cell lines, while MET signaling and an erlotinib signature are enriched in FLCN^POS^ cell lines. Except for the increased MET signaling in FLCN^POS^ cell lines, these results are in line with the above INKA analysis. The apparently conflicting outcome of both analyses regarding MET might be explained by the enhanced phosphorylation of MET inhibitory site Tyr1003, potentially counteracting the effects of MET activation (Tyr1235) in FLCN^NEG^ RPTEC (following section, Fig. 4*F*). Two specifically and significantly enriched signatures in FLCN^POS^ cells are associated with the cellular response to U0126 and CHIR9902, which are inhibitors of MEK1/2 and GSK3/Wnt-pathway, respectively. Furthermore, pSTY PTM-SEA shows enrichment (*p* < 0.1) of AKT1, Angiotensin II, CDK2, Phorbol Ester, PRKAA1, and T-cell receptor signatures in FLCN^NEG^ cell lines.

### Phosphoproteomic Profiling Reveals Hyperphosphorylated Kinases Upon FLCN Loss

To further pinpoint the main effects of FLCN loss on phosphorylation, we decided to focus on hyperphosphorylated kinases as these might contribute most substantially to oncogenic transformation. Based on INKA, we identified EGFR, MET, and EPHA2/EPHB1 as major RTKs that are hyper phosphorylated upon FLCN loss. Figure 4*A* shows EGFR together with its substrates that were identified to be differentially phosphorylated in FLCN^NEG^ RPTECs. To investigate EGFR and its downstream signaling in more detail, we plotted the levels of individual phosphorylation sites of EGFR and significantly differentially phosphorylated EGFR substrates as bar graphs (Fig. 4*B*). For EGFR itself, none of the canonical (auto)phosphorylation sites (*i.e.*, Tyr992, Tyr1045, Tyr1068, Tyr1148, and Tyr1173) (24) were identified in our study. Three different tyrosine phosphorylation sites, Tyr1125, Tyr1138, and Tyr1172, were significantly (*p* ≤ 0.05) more phosphorylated in the absence of FLCN. Currently, the exact biological consequences of these tyrosine phosphorylation sites are not clear, but all three respond to EGF stimulation and share the same interaction partner GRB2 (25). Of all differentially phosphorylated EGFR substrates, epidermal growth factor receptor kinase substrate 8 (EPS8) showed the strongest increase in phosphorylation upon FLCN loss. EPS8 participates in enhancement of EGF-dependent mitogenic signaling, transduction of signals *via* RAC1, and trafficking through RAB5 (26, 27).

EPHA2 together with its (significantly changed) differential substrates are depicted in Figure 4*C*, with individual phosphorylation sites in FLCN^POS^ and FLCN^NEG^ cells shown in bar graphs in Figure 4*D*. Upon FLCN loss, one EPHA2 site located in the sterile alpha motif (SAM) domain (Tyr930) is significantly less phosphorylated, while two sites in the kinase domain (Tyr628 and Tyr694) show higher phosphorylation in FLCN^NEG^ cells, although the latter effects are not significant (*p* > 0.05). The phosphorylation of EPHA2 Tyr930 is regulated by the PTPFR/LAR phosphatase and may play a role in cell migration (28). Phosphorylation of EPHA2 substrates GIT1, BCAR1, INPLL1, and VAV3 is significantly higher in FLCN^NEG^ cells, although differences in normalized intensities are variable.

Another hyperphosphorylated RTK that we explored in more depth is MET. Figure 4*E* shows MET and its downstream substrates detected in our pY data. Bar graphs in Figure 4*F* show differentially phosphorylated sites of MET and its substrates PTK2, CBL, CTNN, and CTTND1. Remarkably, not all MET substrates show higher phosphorylation in FLCN^NEG^ RPTEC cells. Furthermore, only one of the two phosphosites in the activation loop of the kinase domain was significantly more phosphorylated upon FLCN loss (Tyr1235). In addition, the juxtamembrane located autophosphorylation site Tyr1003 was more highly phosphorylated in FLCN^NEG^ cells, possibly promoting ubiquitination and MET degradation *via* CBL (29, 30). CBL is a substrate of MET, and its tyrosine-domain is also phosphorylated at a significantly higher rate FLCN loss. Note that PTK2 and CTNND1 are known substrates of both MET and EGFR (31, 32, 33) and play a role in cell adhesion, migration, proliferation, and a wide variety of signaling transduction pathways. The top 10 of most differential kinases, together with details about specific differential (*p* < 0.05) pY residues, location, and biological effects, are shown in supplemental Table S1.

**Fig. 5:**
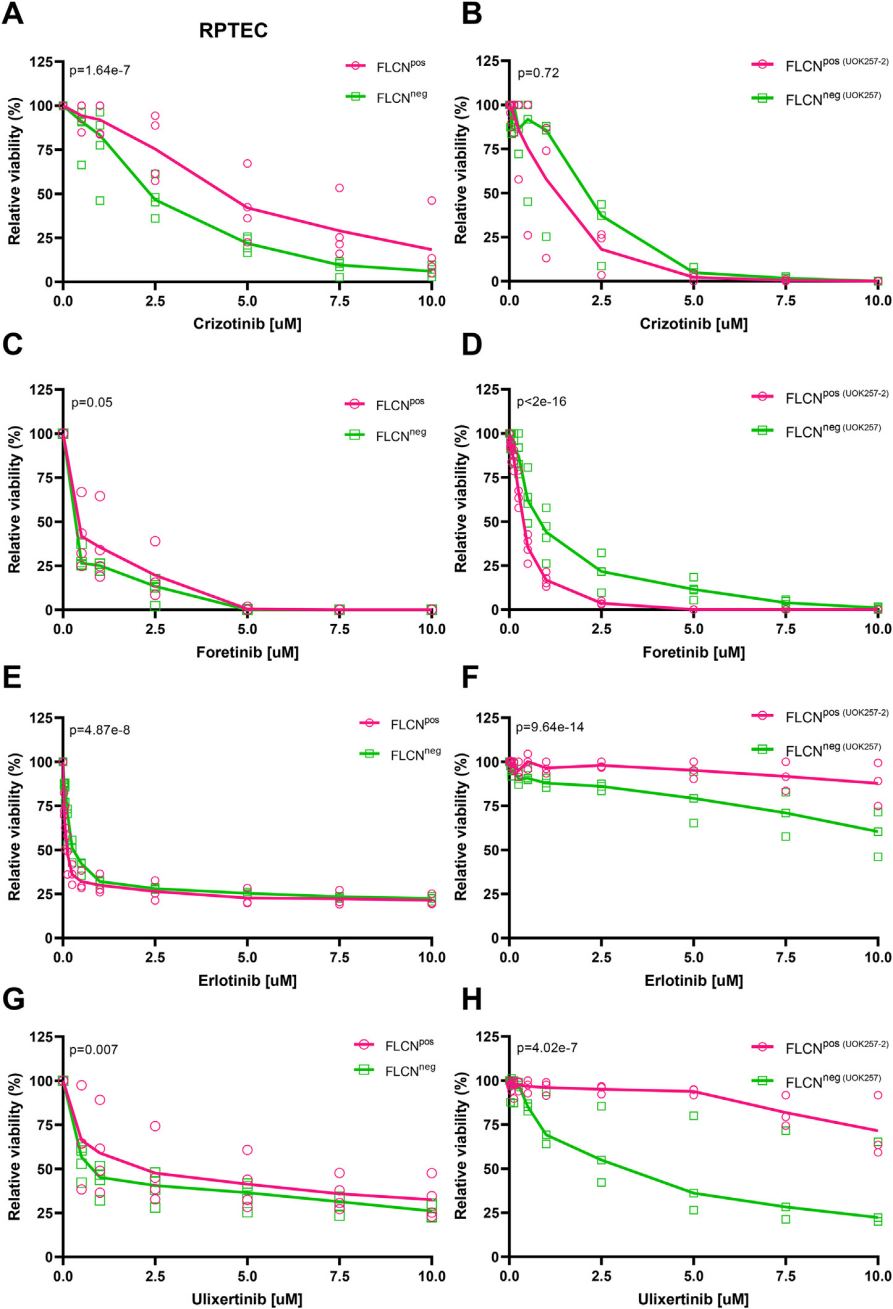
**Tyrosine kinase inhibitor dose–response curves for FLCN**^**POS**^**and FLCN**^**NEG**^**cell lines.***A*, crizotinib dose–response curves (n = 4) of FLCN^POS^*versus* FLCN^NEG^ RPTECs show a difference in sensitivity to MET inhibition (*p* = 1.64e-7). *B*, crizotinib dose–response curves (n = 3) of BHD renal tumor cell line UOK257 (FLCN^NEG^) and the isogenic FLCN reconstituted tumor cell line UOK257-2 (FLCN^POS^) show no difference in sensitivity to MET inhibition (*p* = 0.72). *C*, foretinib dose–response curves (n = 4) of FLCN^POS^*versus* FLCN^NEG^ RPTECs show minor difference in sensitivity to MET inhibition (*p* = 0.05). *D*, foretinib dose–response curves (n = 4) of BHD renal tumor cell line UOK257 (FLCN^NEG^) and the isogenic FLCN reconstituted tumor cell line UOK257-2 (FLCN^POS^) show a difference in sensitivity to MET inhibition (p<2e-16). *E*, erlotinib dose–response curves (n = 3) of FLCN^POS^*versus* FLCN^NEG^ RPTECs show a difference in sensitivity to EGFR inhibition (*p* = 4.87e-8). F. erlotinib dose–response curves (n = 3) of BHD renal tumor cell line UOK257 (FLCN^NEG^) and the isogenic FLCN reconstituted tumor cell line UOK257-2 (FLCN^POS^) show a difference in sensitivity to EGFR inhibition (*p* = 9.64e-14). *G*, ulixertinib dose–response curves (n = 4) of FLCN^POS^*versus* FLCN^NEG^ RPTECs show a difference in sensitivity to MAPK1 inhibition (*p* = 0.007). *H*, ulixertinib dose–response curves (n = 3) of BHD renal tumor cell line UOK257 (FLCN^NEG^) and the isogenic FLCN reconstituted tumor cell line UOK257-2 (FLCN^POS^) show a difference in sensitivity to MAPK1 inhibition (*p* = 4.02e-7). BHD, Birt–Hogg–Dubé; FLCN, folliculin; RPTEC, renal proximal tubular epithelial cell.

As previous studies linked MET activation with the development of both sporadic and hereditary RCC (34, 35), MET might be contributing to oncogenic transformation of FLCN^NEG^ renal cells. As we observed enhanced phosphorylation of MET in FLCN-deficient cells, we subsequently tested whether FLCN^NEG^ cells are more sensitive to MET inhibition than wildtype cells. For our drug-response experiments, we decided to use a diploid FLCN^POS^ and FLCN^NEG^ RPTEC isogenic cell line pair that is, due to an improved synthetic gRNA and Cas9 protein delivery protocol, TP53 wildtype and harbors no Cas9 expression construct (8). To inhibit MET, we tested two kinase inhibitors, Crizotinib and Foretinib, which both bind to the ATP-binding site. These inhibitors have been tested in papillary RCC, which is a specific RCC subtype that often develops in patients harboring (germline) MET mutations (36, 37). Foretinib is a multikinase inhibitor targeting, among others, RET, NRTK1, SRC, LCK, FLT3, and the FLCN-dependent kinase identified in this study, EPHA2 (Fig. 4, *C* and *D*). Crizotinib targets MET, ROS1, and ALK (38, 39). Relative viabilities are depicted as dose–response curves in Figure 5. While we observed that FLCN^NEG^ RPTECs displayed a slight increase in sensitivity to both inhibitors (Fig. 5, *A* and *C*), this effect was not observed when treating patient-derived BHD renal tumor cell line UOK257 (FLCN^NEG^) and the isogenic FLCN reconstituted tumor cell line UOK257-2 (FLCN^POS^) (Fig. 5, *B* and *D*).

As we observed differential phosphorylation of EGFR, even though at the canonical activation sites, we investigated the response to EGFR inhibitor Erlotinib in the same isogenic cell line pairs. Dose–response curves of RPTEC FLCN^POS^ *versus* FLCN^NEG^ are shown in Figure 5*E* and reveal strong general toxicity, already in the nanomolar range. For the isogenic tumor cell line pair UOK257, we observed that FLCN^NEG^ cells were specifically more sensitive to EGFR inhibition than the FLCN-reconstituted cells (Fig. 5*F*). The strong general cytotoxicity of EGFRi as observed in RPTECs was not observed in UOK257 cells.

Since our INKA analysis identified enhanced activity of MAPK1/MAPK3 (ERK2/ERK1 respectively) in FLCN^NEG^ cells we next investigated Ulixertinib as a highly selective MAPK inhibitor of signaling downstream of multiple RTKs, including EGFR, MET, and EPHA2. Ulixertinib dose–response curves of FLCN^POS^ *versus* FLCN^NEG^ RPTECs are depicted in Figure 5*G*. Although Ulixertinib treatments showed some variation between independent experiments, we observed a difference in sensitivity dependent on FLCN expression. The FLCN^NEG^ renal tumor cell line UOK257 appeared to be more sensitive to MAPK1/3 inhibition when compared with FLCN^POS^ UOK257-2 (Fig. 5*H*). Statistically significant differences in viability of FLCN^POS^ and FLCN^NEG^ cells were calculated and *p*-values are indicated for each graph. Combination treatments of MAPK inhibition with MET inhibition or EGFR inhibition did not reveal a stronger effect than MAPK inhibition alone (data not shown).

To further elucidate the differences in response to kinase inhibitors between FLCN^POS^ and FLCN^NEG^ RPTEC and UOK257 cells, we performed comprehensive phosphoproteomic profiling of the UOK257 (FLCN^NEG^) and the isogenic FLCN reconstituted tumor cell line UOK257-2 (FLCN^POS^). In supplemental Fig. S6, *A* and *B*, supervised hierarchical cluster analyses of FLCN differential phosphopeptide intensities are shown. Similar to our comparisons in RPTEC, this revealed a clear separation between the two groups, indicating that numerous phosphorylation events are directed by FLCN inactivation. Next, we performed INKA analysis, and in supplemental Fig. S6*C*, the top 20 of most active kinases identified in UOK257 and UOK257-2 (in both pY and pSTY datasets) are shown.

Importantly, EGFR, MET, EPHA2, MAPK1, MAPK3, and RPS6 kinase pathways were strongly dependent on FLCN in RPTEC and also strongly respond to FLCN reconstitution in this BHD tumor cell line, as highlighted in the UOK257 INKA rankings in orange (supplemental Fig. S6*C*). To further look into the overlap of activated kinases upon FLCN loss, we made Venn diagrams of kinases with the highest activities (INKA score >75) in RPTEC and UOK257 cell lines (supplemental Fig. S6*D*). These show that, based on pY data, MET, EPHA2, GSK3B, and PTK2 were the predominant, overlapping, active kinases, while based on pSTY data, MAPK1, MAPK3, and CDK1 were overlapping active kinases. FLCN-specific activities of kinases in UOK257 and UOK257-2 that are also top differential kinases dependent on FLCN in RPTEC are shown as box plots in supplemental Fig. 6*E*. Activities of EPHA2, EPHB1, RPS6KA1, RPS6KA3, MAPK1, MAPK3, and PAK1 are increased by FLCN loss in both cell line backgrounds, and we also confirmed a decrease in CDK1/2, PTK2, and MAPK10 activities upon FLCN loss in both cell line backgrounds (overlap is indicated in orange in supplemental Fig. S6*E* and by asterisks in Fig. 2). In contrast to RPTEC, global EGFR and MET activities showed less activity in the FLCN-negative UOK257 tumor cell line when compared with the FLCN-restored UOK257-2, even though EGFR and MET emerge as significantly important pathways in each cell model.

Normalized intensities of individual phosphosites detected in UOK257 datasets, related to the major kinases dependent on FLCN in RPTEC, are shown as bar graphs in supplemental Fig. 6*F*. Summarizing, we found that the higher phosphorylation of MET_Tyr1235, MAPK1_Thr185, canonical MAPK3 activation sites Thr202 and Tyr204, and RPS6KA1_Ser380 in FLCN^NEG^ UOK257 cells was concordant with our findings in RPTEC. Taken together, these phosphoproteomic data support the differential sensitivities of RPTEC and UOK257 cells to kinase inhibitors and point toward a role for FLCN in MAPK1/3 and downstream RPS6 kinase signaling.

### FLCN Loss Regulates MAPK Signaling and Phosphorylation of TFEB

**Fig. 6:**
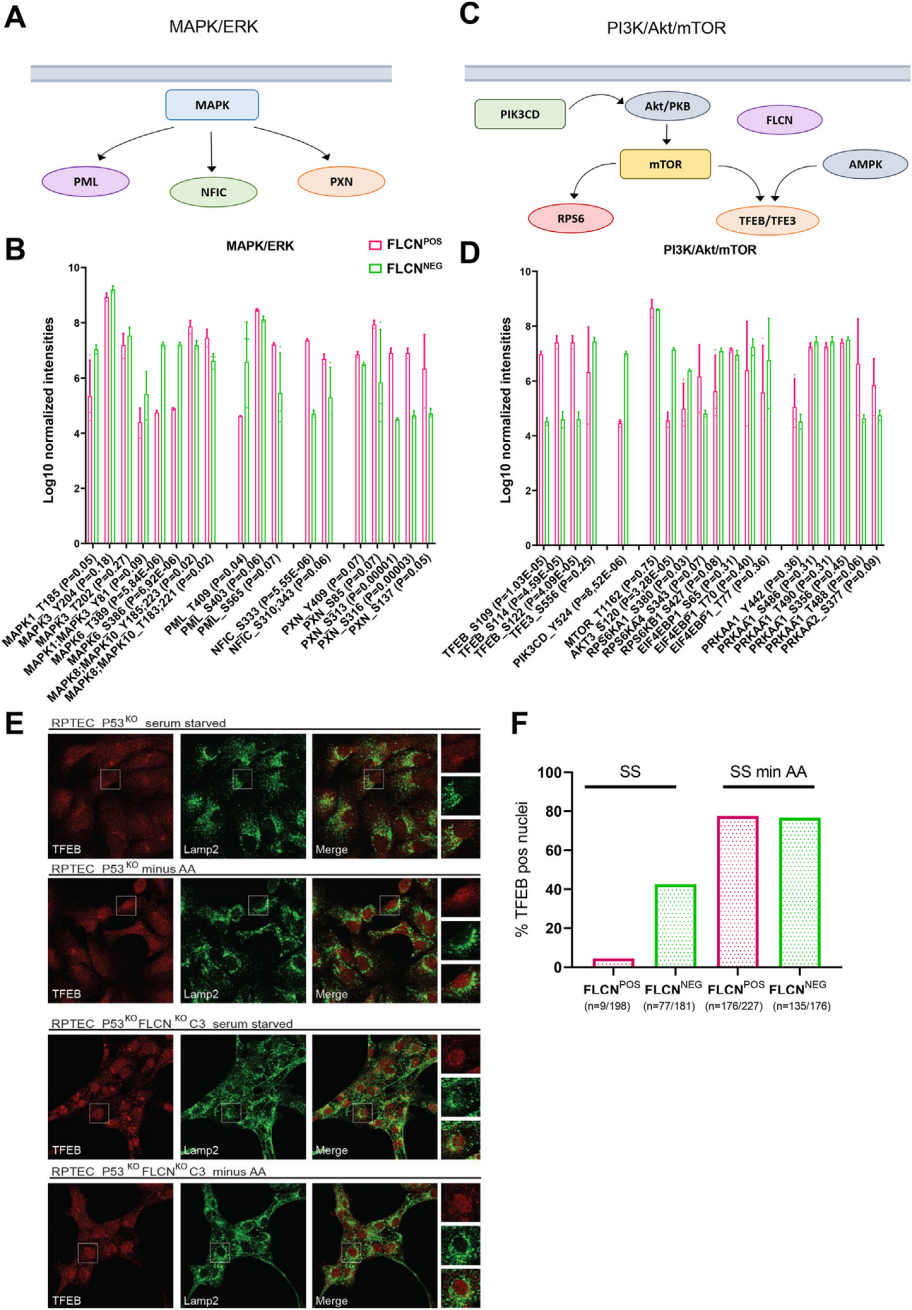
**Loss of FLCN regulates MAPK signaling and TFEB phosphorylation and localization.***A*, MAPK together with its substrates that were identified to be differentially phosphorylated upon FLCN loss in RPTECs. *B*, bar graph of normalized phosphosite intensities of MAPK1/3/6/8/10 and substrates PML, NFIC, and PXN. *p*-values are indicated for each phosphosite depicted in the bar graph. *C*, PI3K/Akt/mTOR together with their substrates that were identified to be differentially phosphorylated upon FLCN loss in RPTECs. *D*, bar graph of normalized phosphosite intensities of PI3K/Akt/mTOR signaling, as these are known upstream regulators of ribosomal S6 kinases. *p*-values are indicated for each phosphosite depicted in the bar graph. *E*, immunofluorescence costaining of TFEB and lysosomal marker LAMP2 show enhanced nuclear TFEB upon amino acid starvation of FLCN^POS^ RPTECs. Upon FLCN loss, TFEB localization is nuclear independent of nutrient availability. AA, amino acids. *F*, quantification of TFEB-positive nuclei in FLCN^POS^ and FLCN^NEG^ RPTECs in either serum-starved (SS) medium or in medium depleted of both serum and amino acids (SS min AA). FLCN^NEG^ show nuclear TFEB in the presence of amino acids, while FLCN^POS^ show cytoplasmic TFEB as expected in a fed condition. FLCN, folliculin; RPTEC, renal proximal tubular epithelial cell; TFEB, transcription factor EB.

The INKA analyses of pSTY indicated MAPK, HIPK2, and RP6S kinases as the most differential kinase pathways upon FLCN loss in RPTECs. Figure 6*A* shows MAPK and its differentially regulated substrates PML, NFIC, and PXN. MAPK1_Thr185 and MAPK6_Thr389 and Ser386 were all significantly more phosphorylated upon FLCN loss, whereas the canonical activation sites MAPK8;MAPK10_Tyr185;223 and Thr183;221 were significantly less phosphorylated. Note that the significantly differential substrate sites (NFIC_Ser333 and PXN_Ser313, Ser316 and Ser137) are less phosphorylated in FLCN^NEG^ RPTEC, except PML_Thr409 (Fig. 6*B*). For HIPK2 and its substrates HMGA1 and TRIM28, we observed that HIPK2_Ser827 became phosphorylated upon FLCN loss, while sites identified in its substrates were phosphorylated at significantly lower levels (supplemental Fig. S7*A*). HIPK2, HMGA1, and TRIM28 have been linked to renal development, fibrosis, and cancer (40, 41, 42, 43, 44). To test whether FLCN knockout cells are dependent on HIPK2, we treated RPTEC with the HIPK2/CK2 inhibitor Silmitasertib but did not detect differences in sensitivity between FLCN^POS^ and FLCN^NEG^ cell lines (supplemental Fig. S7*B*).

In Figure 6*C*, we show several canonical components of PI3K/Akt/AMPK/mTOR signaling, as these are known upstream regulators of the RPS6 kinase and protein (33). Phosphorylation of both PIK3CD_Tyr524 and Akt3_Ser120 was strongly enhanced in FLCN^NEG^ cell lines (Fig. 6*D*). Upon FLCN loss, we did not detect differential phosphorylation of Akt1/2 and downstream mTOR/EIF4EBP1 phosphosites, and no significant change in the phosphorylation of AMPK subunits PRKAA1 and PRKAA2 was observed. The top 10 of most differential kinases, together with details about specific differential (*p* < 0.05) pSTY residues, location, and biological effects, are shown in supplemental Table S1. These results indicate that, at least in RPTEC, AMPK and mTOR are not the main effectors of FLCN signaling. Interestingly, in line with a role for TFEB in kidney tumorigenesis in a BHD mouse model (45), we identified significant dephosphorylation of TFEB_Ser109, Ser114, and Ser122 upon FLCN loss.

TFEB and its close family member TFE3 are transcription factors directing expression of lysosomal and autophagy genes under growth restrictive conditions (46, 47, 48). To further explore the regulation of TFEB by FLCN, we performed immunofluorescent costainings of TFEB and lysosomal marker LAMP2 in RPTEC P53^KO^ (FLCN^POS^) and P53^KO^ FLCN^KO^ C3 (FLCN^NEG^) cells in the presence or absence of amino acids (AA), as shown in Figure 6*E*. FLCN^POS^ cells showed diffuse TFEB localization under serum-starved conditions but enhanced translocation to the nucleus after the withdrawal of amino acids (upper panels). In contrast, we observed increased nuclear localization of TFEB in FLCN-deficient cells even in the presence of amino acids, revealing that FLCN^NEG^ cells fail to convey proper regulation of TFEB. For quantification of TFEB immunofluorescent stainings, percentages of cells with nuclear TFEB were calculated and are shown in Figure 6*F*.

The decrease of TFEB phosphorylation was also observed in immunoblots of total TFEB, with a clear downward mobility shift on SDS-PAGE after amino acid starvation of wildtype cells, similar to the migration of TFEB derived from FLCN^NEG^ cells (supplemental Fig. S7*C*). Furthermore, in concordance with the strong nuclear localization of TFE3 observed in previous studies (8, 49, 50), we found that TFE3 also shifts downward on Western blots, indicating dephosphorylation, in response to amino acid starvation or FLCN loss in RPTECs (supplemental Fig. S7*C*). In addition, to confirm specificity of the TFEB antibody used for immunofluorescence and immunoblotting, the results of siTFEB experiments are shown in supplemental Fig. S7, *D* and *E*.

**Fig. 7:**
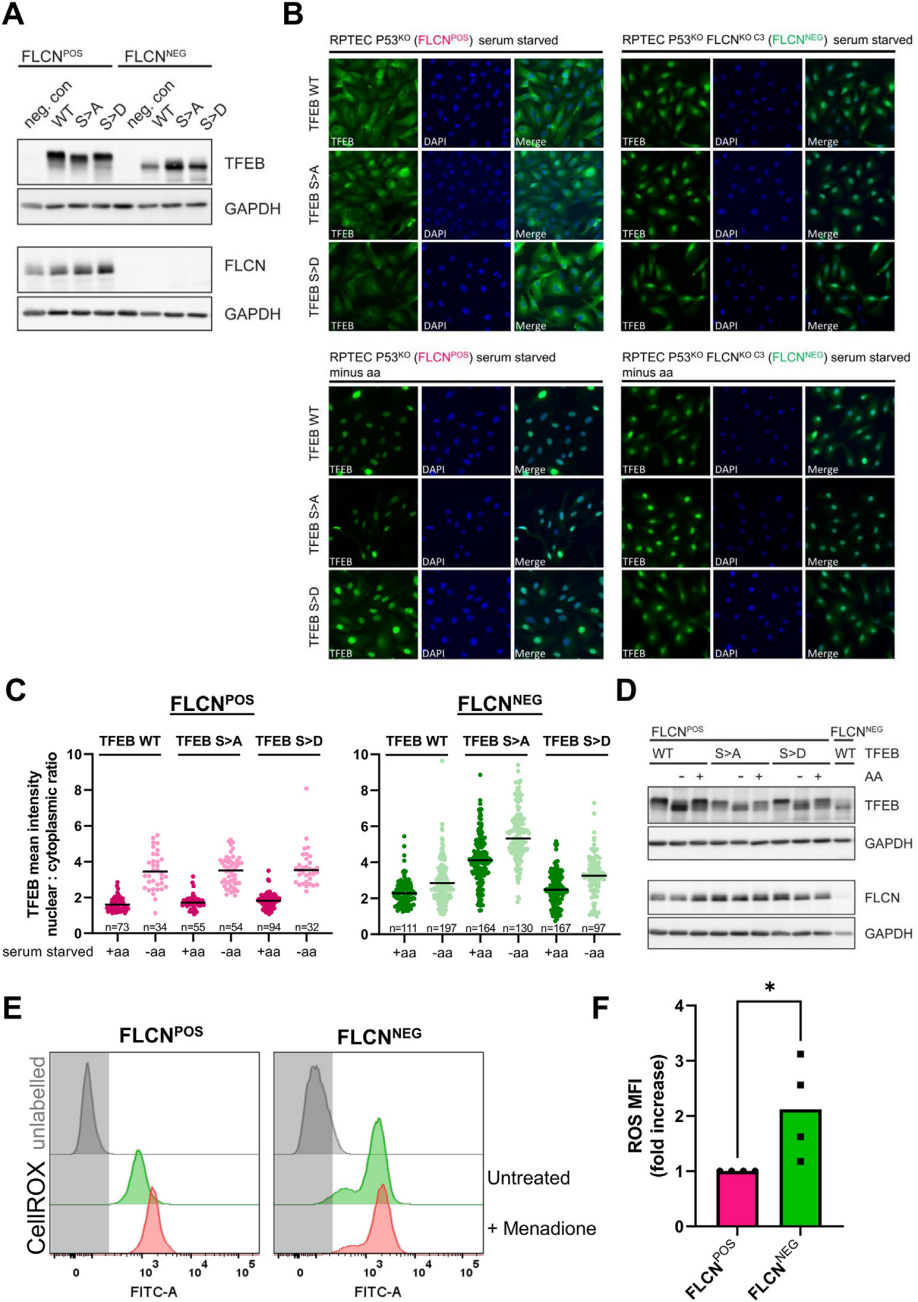
**FLCN-sensitive phosphoserines modulate nucleocytoplasmic shuttling of TFEB.***A*, Western blot of TFEB in FLCN^POS^ and FLCN^NEG^ RPTECs that overexpress WT, S>A, or S>D phosphomutant TFEB. FLCN protein was absent in FLCN^NEG^ cells and GAPDH was used as a loading control. *B*, representative photos of immunofluorescent stainings of FLCN^POS^ and FLCN^NEG^ RPTECs that overexpress WT, S>A, or S>D phosphomutant TFEB in serum-starved medium or in medium depleted of both serum and amino acids (minus aa). Stainings were performed with FLAG antibody to ensure that the signal of endogenous TFEB could not interfere with the results. Quantifications are shown in Figure 7*C*. *C*, quantifications of immunofluorescent stainings (shown in Fig. 7*B*) of FLCN^POS^ and FLCN^NEG^ RPTECs that overexpress WT, S>A, or S>D phosphomutant TFEB in serum-starved medium −/+ amino acid starvation. Nuclear:cytoplasmic ratios of mean TFEB intensities were calculated based on automated image analyses. For each condition, the total number of cells analyzed is indicated and means are shown as *black bars*. *D*, Western blot of TFEB in FLCN^POS^ RPTECs TFEB phosphomutants shows clear downward mobility shifts on SDS-PAGE upon amino acid (AA) starvation. In FLCN^NEG^ RPTECs this downward mobility shift is, even in the presence of amino acids, more abundant, which points toward additional dephosphorylation events. GAPDH was used as a loading control. *E*, flow cytometry histograms of fluorescent intensities of FLCN^POS^ and FLCN^NEG^ RPTECs stained for intracellular reactive oxygen species (ROS) with the CellROX reagent. As a positive control a Menadione treated (100 μM, 1 h) sample was included. Fold changes of independent experiments were calculated and are shown as a bar graph in Figure 7*F*. *F*, relative ROS levels are shown as fold change in mean fluorescent intensity (MFI) as measured by flow cytometry. FLCN^NEG^ cells show 2.2-fold higher ROS levels than FLCN^POS^ cells (n = 4). FLCN, folliculin; RPTEC, renal proximal tubular epithelial cell; TFEB, transcription factor EB.

The differentially phosphorylated TFEB serines (Ser109, 114, and 122) identified here are identical to those reported to be dephosphorylated in response to oxidative stress (19). To further investigate the regulation of these three phosphoserine sites, we created RPTEC FLCN^POS^ and FLCN^NEG^ cell lines that overexpress a FLAG-tagged version of TFEB wildtype (WT) or TFEB phosphomutants (serine to alanine, S109A, S114A, S122A; in short S>A, and serine to aspartic acid, S109D, S114D, S122D; in short S>D). In Figure 7*A*, overexpression of TFEB was validated by Western blot. Remarkably, both TFEB WT and phosphomutants present in cell lysates of FLCN^NEG^ cells migrated faster, indicating that TFEB in FLCN^POS^ and FLCN^NEG^ RPTEC cells differ in additional posttranslational modifications. Next, we performed immunofluorescent stainings to assess the effects of S>A (mimicking dephosphorylation) and S>D (mimicking phosphorylation) at TFEB Ser109, Ser114, and Ser122 sites in a FLCN^POS^ and FLCN^NEG^ background. Similar to endogenous TFEB (Fig. 6*E*), overexpressed TFEB WT was more nuclear upon FLCN loss (Fig. 7*B*, upper panels) or amino acid starvation (Fig. 7*B*, lower panels). In the presence of amino acids, we observed no significant increase of FLCN^POS^ cells with nuclear TFEB as a result of the S>A phosphomutant, while mutation of these three serines further enhanced nuclear TFEB localization in FLCN^NEG^ cells. The S>D phosphomutant behaved as TFEB WT and did not restore cytoplasmic TFEB localization in FLCN^NEG^ cells. This indicates that one or more additional modifications normally retaining TFEB in the cytoplasm are lost in FLCN deficient cells. Upon starvation of amino acids, we observed strong nuclear localization of TFEB in WT, S>A, and S>D cells, independent of FLCN status. Quantifications of TFEB localization in WT, S>A, and S>D serum-starved cells, in the presence or absence of amino acids, are shown in Figure 7*C*.

Together, this analysis reveals a clear increased nuclear localization of all TFEB isoforms in response to amino acid starvation in FLCN^POS^ cells. In FLCN^NEG^ cells, the basal level of nuclear TFEB is clearly increased. Although nuclear TFEB accumulates further upon amino acid depletion in FLCN-deficient cells, the response is weaker compared with that of FLCN-proficient cells. Interestingly, the fact that the TFEB S>A phosphomutant is still responsive to amino acid starvation (Fig. 7*C*) and refeeding (Fig. 7*D*) in the presence of FLCN suggests a role for additional TFEB posttranslational modifications to convey a complete nutrient starvation response.

These observations are in agreement with the hypothesis that, upon FLCN loss, a cellular stress response is induced that leads to partial dephosphorylation and enhanced nuclear localization of TFEB. Complete dephosphorylation of TFEB Ser109, Ser114, and Ser122, as mimicked by the S>A phosphomutant, appears to drive TFEB further into the nucleus in the absence of FLCN.

To see if the dephosphorylation of Ser109, Ser114, and Ser122 could be related to the presence of oxidative stress, we next assessed whether there is an increase in ROS in FLCN^NEG^ RPTECs. Representative flow cytometry histograms of fluorescent intensities of FLCN^POS^ and FLCN^NEG^ RPTECs stained for intracellular ROS are shown in Figure 7*E* (green histograms). Fold changes of independent experiments were calculated and are shown as a bar graph in Figure 7*F*. Based on the mean of these experiments, we conclude that FLCN-deficient RPTECs exhibit a significant ±2-fold increase in ROS. As a positive control, treatments with ROS-inducing agent Menadione (100 μM for 1 h) showed a clear increase in ROS in FLCN^POS^ cells (red histograms). We thus propose that RPTECs experience oxidative stress upon FLCN loss, which may contribute to TFEB dephosphorylation.

We did not identify a kinase of TFEB Ser109, Ser114, and Ser122 that might be inhibited by FLCN loss. Our INKA analysis revealed some kinases that are less active upon FLCN loss, such as CDK1/2 (pY), MAPK10, PRKCA, and ROCK2 (pSTY). In our previous study we identified reduced growth rates of FLCN^NEG^ cells, and we therefore looked further into differential phosphorylation of canonical cell cycle regulators. In supplemental Fig. S7*E*, we show identified phosphosites of CDK1, CDK2/3, and its inhibitory kinase WEE1 (51) which were not significantly different. INKA identified lowered CDK1/2 activity based on other FLCN-dependent CDK substrates, such as NPM, TMPO, RB1, TOP2B, and LMNA (all *p* < 0.01). Although we cannot rule out that the decrease of TFEB phosphorylation in FLCN-deficient cells is secondary to cell cycle effects and reduced CDK2 activity, we consider it more likely that a kinase or phosphatase directly downstream of FLCN directs the TFEB phosphorylation state.

In addition, INKA revealed that ROCK1 and ROCK2, key regulators of actin cytoskeleton and cell polarity, and their substrates CFL1 and CFL2 are less active in FLCN^NEG^ cells. In supplemental Fig. S7*F* we depicted individual phosphosites as bar graphs, showing that phosphorylation of ROCK2_Ser1137 and CFL1_Thr70 was reduced upon FLCN loss.

To summarize, in this study we identified that FLCN loss elevates phosphorylation of numerous kinases, including tyrosine kinases EGFR, MET, and EPHA2/B1, as well as their substrates EPS8, CBL, and BCAR1 and downstream targets MAPK1/3 and RPS6K. Dose–response curves of MET and EGFR inhibitors did not indicate a strong dependency on FLCN in RPTEC but revealed that BHD renal tumor cell line UOK257 is reliant on EGFR and MAPK1/3 signaling. Phosphoproteomic analyses of the BHD renal tumor cell line UOK257 and FLCN-reconstituted UOK257-2 cells confirmed a role for FLCN in MAPK1/3 and downstream RPS6K1/3 signaling.

Finally, our phosphoproteomic analysis uncovers Ser109, Ser114, and Ser122 TFEB sites as targets of FLCN signaling in renal cells. Upon FLCN loss, these TFEB phosphoserines are dephosphorylated, which may be explained by the observation that FLCN^NEG^ cells experience oxidative stress.

**Fig. 8:**
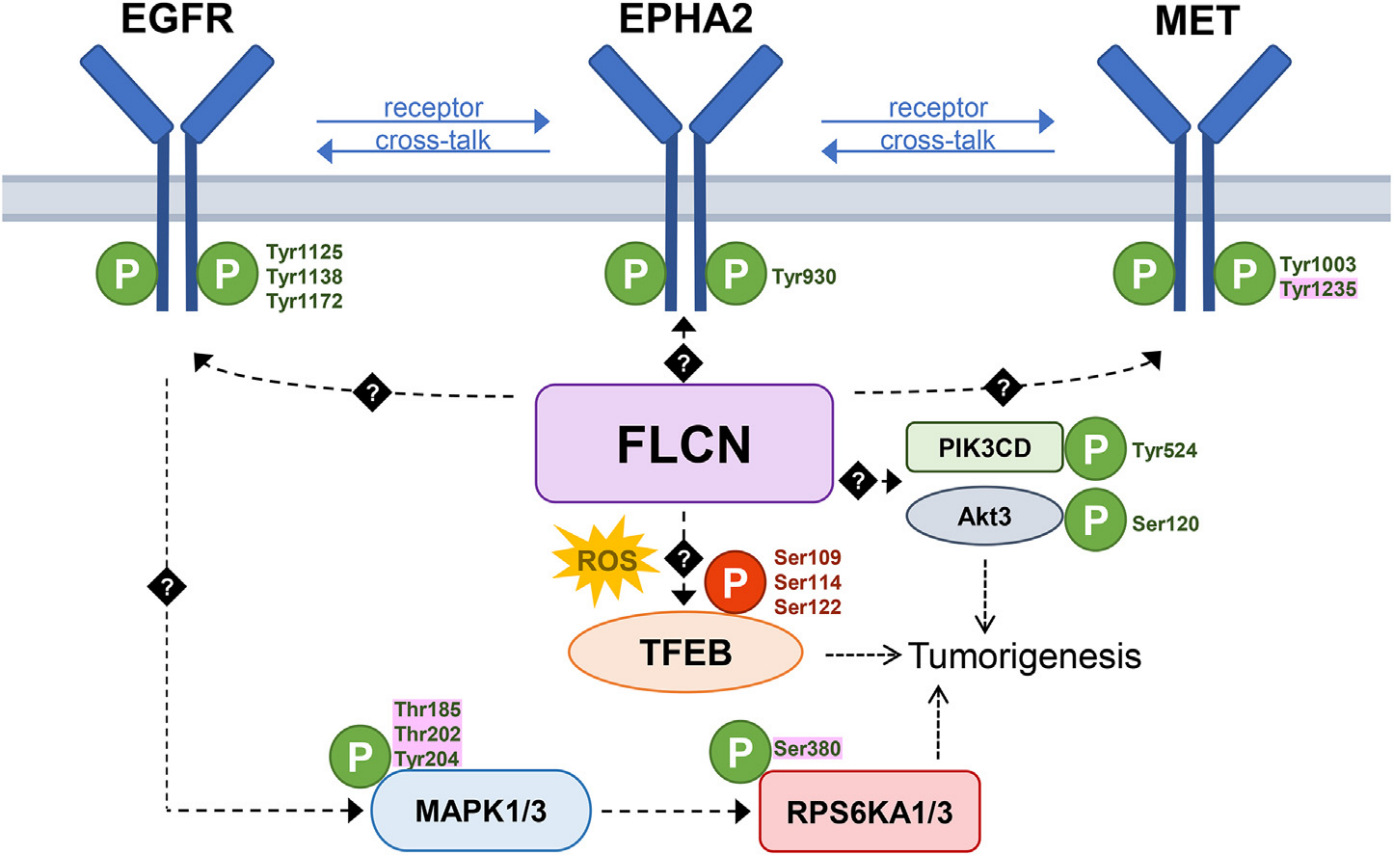
**Schematic overview of differential phosphorylation pathways upon FLCN loss.** A scheme portraying most dominant pathways where FLCN is involved in protein and receptor phosphorylation in our renal epithelial cell model. *Dashed lines* show where FLCN is involved, either directly or indirectly *via* (multiple) unknown other kinases as indicated by *blackdiamond-shaped squares* with question marks. Highlighted in *pink* are specific phosphosites that are differentially phosphorylated upon FLCN deficiency in both renal proximal tubular epithelial cells and UOK257 cell lines. As TFEB is dephosphorylated upon FLCN loss, we marked these specific differential phosphosites *red*. FLCN, folliculin; ROS, reactive oxygen species; TFEB, transcription factor EB.

In Figure 8, we summarize these findings and schematically visualize the most dominant pathways where FLCN is involved in protein and receptor phosphorylation in our renal epithelial cell model.

## Discussion

To gain more insight into how FLCN acts to suppress renal tumorigenesis in BHD syndrome, we have investigated the role of FLCN in regulating cellular signaling pathways *via* protein and receptor phosphorylation by determining complete phosphoproteomic profiles. We used renal epithelial cell lines we previously developed to explore FLCN-dependent gene and protein expression changes (8). Although our *in vitro* human renal tubular cell models may not fully recapitulate the effect of FLCN deletion *in vivo*, we found clear differential tyrosine, serine, and threonine phosphorylation events upon FLCN loss in renal epithelial cells that might play a role in precancerous cellular changes.

Previously, we discovered two transcriptional programs that were induced upon FLCN loss. In agreement with the induction of the E-box transcriptional gene expression signature upon FLCN loss, in this study we found that phosphorylation of TFEB Ser109, Ser114, and Ser122 is dependent on FLCN. In FLCN^NEG^ cells, these sites are strongly dephosphorylated, which correlated with increased nuclear localization, activation of TFEB, and induction of E-box genes such as RagD. Transcriptional activation of TFEB and the RagC/RagD GTPases has been shown to control mTORC1 activity and drive the development of kidney abnormalities in a BHD mouse model (45, 52). In two recent studies (53, 54), the structure of the FLCN-FNIP2-Rag-Ragulator complex was revealed, identifying the mechanism for Rag GTPase activity with FLCN Arg164 as the catalytic residue for the GAP activity toward RagC.

An important novel finding regarding FLCN-loss dependent TFEB phosphorylation is that we find significant dephosphorylation of TFEB Ser109, Ser114, and Ser122. These three phosphoserine sites on TFEB were previously described to be dephosphorylated upon Torin-1 treatment or in response to oxidative stress by PP2A (19). Indeed, we found ROS levels significantly increased in our FLCN^NEG^ RPTEC cells, a finding that is consistent with a recent observation in MCF7 cells (55). Furthermore, gene set enrichment analyses of our previous transcriptomic and proteomic study of FLCN^NEG^ RPTEC cells revealed an enrichment of oxidative phosphorylation and ROS hallmark gene sets (8). Using phosphomimetic and phosphodead mutants of these serine residues, we find that these sites have a modulatory role in nucleocytoplasmic shuttling of TFEB and cannot fully explain the nuclear translocation of TFEB in FLCN^NEG^ cells. This situation is reminiscent of FOXO transcription factors, whose localization can be modulated by oxidative stress (56). Additional changes in posttranslational modifications of TFEB as a result of FLCN loss are clear from increased mobility of TFEB and mutant versions studied here on SDS-PAGE.

Obviously, mTOR activity may play a role in these changes as it is known that mTOR directly phosphorylates TFEB on Ser122, Ser138, Ser142, and Ser211 (33, 57, 58) and based on its motif specificity, mTOR could be the responsible kinase for phosphorylation of TFEB Ser109 and Ser114 too (19, 59). Ser138 and Ser142 are hierarchically phosphorylated to control CRM1-mediated nuclear export of TFEB (58), while phosphorylation of Ser211 in cooperation with Ser122 plays a critical role in the regulation of TFEB nuclear localization as well (57). Unfortunately, in our phosphoproteomic analyses, we did not detect TFEB Ser138, Ser142, and Ser211 phosphopeptides or equivalent phosphosites of TFE3. This is in agreement with a strong underrepresentation of these sites in other, publicly available, phosphoproteomic datasets (33). Therefore, we do not know which of these phosphosites are differentially phosphorylated upon FLCN loss in our cell model.

Hypophosphorylated TFEB may seem at odds with previous studies, including our own, that reported increased or normal levels mTORC1 activity upon FLCN loss. Nevertheless, nuclear TFEB is also found in TSC-deficient cells with enhanced mTORC1 activity (60). This abnormal localization can be reverted by overexpression of mutant RagC, demonstrating the critical role that FLCN *via* RagC regulation plays in selecting TFEB as a substrate for mTORC1 (53, 54, 61). However, a previous study by Petit and colleagues (62) showed that FLCN loss results in reduced mTORC1 activity and a decrease in phosphorylation of TFEB Ser211. Also, active mTOR may promote the cytoplasmic retention of TFEB to negatively regulate cellular catabolic processes and autophagy (46, 47, 48), arguing against a role for mTOR activation downstream of FLCN loss.

Taken together, our phosphoproteomic analysis uncovers three TFEB phosphoserine sites as targets of FLCN signaling in renal cells and is in agreement with the emerging picture that FLCN plays a role in maintaining phosphorylation of the E-box transcription factors TFEB and TFE3 (45, 49, 50). Future research will be required to unravel how other TFEB phosphosites contribute to the cellular localization of TFEB in FLCN-deficient cells.

Although we detected higher phosphorylation of the catalytic subunit of PIK3CD upon FLCN loss, the downstream effect of this modification is not fully understood. We did not observe differential downstream phosphorylation of canonical Akt1/2 and mTOR activation sites, nor differences in AMPK phosphorylation upon FLCN loss. We thus hypothesize that, in renal epithelial cells, kinases other than mTOR also play a role in the phosphorylation and regulation of TFEB (63). Interestingly, the specific TFEB serine sites that we found to be differential upon FLCN loss here are Serine/Proline (SP) sites, which are typical substrates of the MAPK and CDK families of kinases (19, 64, 65). Hypothetically, MAPK10 (JNK3) may play a role in TFEB phosphorylation, as the activity of this MAPK appeared to be lowered upon FLCN loss in both RPTEC and UOK257 cell lines (Figs. 2 and S6*E*), while MAPK1/3/6 and 8 activities were higher.

Earlier, we discovered that FLCN loss induced a strong interferon response signature in RPTEC but were not able to link this to canonical activation of the JAK/STAT pathway. Here, our INKA analysis revealed JAK2 as an active kinase in 2/3 FLCN^NEG^ cell lines; however, the only identified differential phosphosite Tyr570 (*p* = 0.08) is known to be an inhibitory site of JAK2 (66, 67). Moreover, we detect a 4.5-fold increase in STAT1_Tyr701 phosphorylation, although in our previous study we did not detect enhanced phosphorylation of this phosphosite by Western blot or enhanced nuclear localization of STAT1. Therefore, it remains to be elucidated how STAT1 is activated by FLCN loss.

Unbiased analyses of our phosphoproteomic data, using INKA and phosphosite enrichment analyses, revealed that phosphorylations of kinases and substrates within multiple biological pathways are clearly FLCN-loss dependent. The enhanced phosphorylation of EGFR, MET, and EPHA2/B1 upon FLCN loss is interesting in light of previous studies that linked these kinases to renal cancer. EPHA2 has been connected to proliferation, drug resistance, and metastatic potential of RCC (68, 69, 70). Also, there is cross talk between the EPHA2 and EGFR/MET receptors (71, 72), and EPHA2 has been shown to play a role in regulation of Rac/Rho GTPases (73, 74), cell migration, and invasion *via* Akt (75). As we also found differential phosphorylation of specific ROCK2, CFL, and EPS8 phosphosites upon FLCN loss, we speculate that loss of FLCN may perturb cell migration and invasion, cytoskeletal organization, and cell polarity of renal epithelial cells, which may be the first steps of oncogenic transformation.

Although none of the well-described EGFR activation phosphorylation sites were identified to be dependent on FLCN, three specific tyrosine phosphorylation sites (Tyr1125, Tyr1138, and Tyr1172) were phosphorylated at significantly higher levels in the absence of FLCN. In tumor cell lines, the loss of FLCN resulted in slower endocytic trafficking of EGFR by decreased Rab7A GTP-to-GDP turnover, resulting in prolonged and elevated phosphorylated EGFR and downstream signaling (76). Comparing our phosphoproteomic data of FLCN^NEG^ renal epithelial cells with that study, we do detect an increase in phosphorylation of ERK1/MAPK3 (Thr202/Tyr204) but do not detect differential phosphorylation of Akt (Ser473) and STAT3 (Tyr705) downstream of EGFR. In addition to EGFR, the reported increase in MET signaling in FLCN-deficient cells is in line with our results.

The substantial overlap within EGFR and MET signaling, and the fact that MET activation has been linked with the development of RCC (34, 35), prompted us to investigate whether FLCN^NEG^ cells were more sensitive to MET inhibition. Although we saw a minor FLCN-dependent effect of MET inhibitors in RPTEC, these effects were not reproducible in BHD tumor cell lines UOK257 and FLCN reconstituted UOK257-2. It could be that the enhanced phosphorylation of the MET inhibitory site Tyr1003 upon FLCN loss counteracts the effects of MET activation (Tyr1235) in RPTEC. Possibly, the metastatic BHD RCC cell line has lost a measurable dependency on MET signaling and may have evolved additional ways to activate downstream components of this pathway, such as MAPK, to drive survival and proliferation. This would also explain why treatments with Ulixertinib revealed that FLCN^NEG^ cells were more sensitive to MAPK1/3 inhibition than FLCN^POS^ cells. These findings warrant preclinical investigations in mouse models testing the effectiveness of Ulixertinib to inhibit BHD tumor growth.

When comparing the phosphoproteomic profiles of our *in vitro* model for BHD syndrome with phosphoproteomic studies done for sporadic RCC, we observed an overlap in activation of specific kinases. A recent study of Van Beijnum and colleagues (77) found that MET, EPHA2, PTK2, EGFR, and Src were among the top-ranked active kinases in five profiled RCC cell lines (786-O, A498, Caki-1, Caki-2, and ACHN). However, none of these renal cancer cell lines harbored a mutation in FLCN (78). A larger proteogenomic characterization study of clear cell RCC *versus* normal adjacent renal tissue revealed activation of EGFR, MAPK/ERK, and Akt/mTOR signaling pathways in renal tumor tissues (79). Apart from EGFR, not many other tyrosine kinases were found in that study, as separate tyrosine-phosphoproteomic profiling was lacking. Although we cannot rule out that EGFR and MET signaling contribute to tumorigenesis of BHD tumors *in vivo*, our *in vitro* findings do not warrant a clinical investigation of EGFR or MET inhibitors as a targeted therapeutic approach.

Taken together, our study provides a comprehensive overview of FLCN-dependent phosphorylation events and yields new insights into renal tumorigenesis, which may help design targeted treatments for patients with BHD.

## Data Availability

Mass spec data are deposited on ProteomeXchange under accession numbers PXD025798 (RPTEC) and PXD030237 (UOK257).

## Supplemental data

This article contains supplemental data (80).

## Conflict of interest

The authors declare that they have no conflicts of interest with the contents of this article.

## Supplemental Data

Supplemental_figures

Files_compliance reviewer
