# Supplementary material for: Phosphoproteomic Analysis of FLCN Inactivation Highlights Differential Kinase Pathways and Regulatory TFEB Phosphoserines
Source: Mol Cell Proteomics. 2022 Jul 19;21(9):100263. doi: 10.1016/j.mcpro.2022.100263 (PMC9421328; doi:10.1016/j.mcpro.2022.100263)
Supplement: Supplemental_figures [file mmc3.pdf]

Supplementary material related to

Phosphoproteomic analysis of FLCN inactivation highlights differential kinase pathways and regulatory TFEB phosphoserines

Iris E. Glykofridis[1], Alex A. Henneman[2], Jesper A. Balk[1], Richard Goeij-de Haas[2], Denise Westland[3], Sander R. Piersma[2], Jaco C. Knol[2], Thang V. Pham[2], Michiel Boekhout[3], Fried J.T. Zwartkruis[3], Rob M.F. Wolthuis[1*] & Connie R. Jimenez[2*]

[1] Amsterdam UMC, location VUmc, Vrije Universiteit Amsterdam, Human Genetics, Cancer Center Amsterdam, De Boelelaan 1118, 1081 HV Amsterdam, The Netherlands
[2] Amsterdam UMC, location VUmc, Vrije Universiteit Amsterdam, Medical Oncology, Cancer Center Amsterdam, De Boelelaan 1118, 1081 HV Amsterdam, The Netherlands
[3] University Medical Center Utrecht, Center for Molecular Medicine, Molecular Cancer Research, Universiteitsweg 100, 3584 CG Utrecht, The Netherlands

* corresponding authors: c.jimenez@amsterdamumc.nl & r.wolthuis@amsterdamumc.nl

Supplementary Figures 1- 7 including legends

Reference

1. Goedhart J, Luijsterburg MS. *VolcaNoseR is a web app for creating, exploring, labeling and sharing volcano plots.* Sci Rep. 2020 Nov 25;10(1):20560. doi: 10.1038/s41598-020-76603-3. PMID: 33239692; PMCID: PMC7689420.

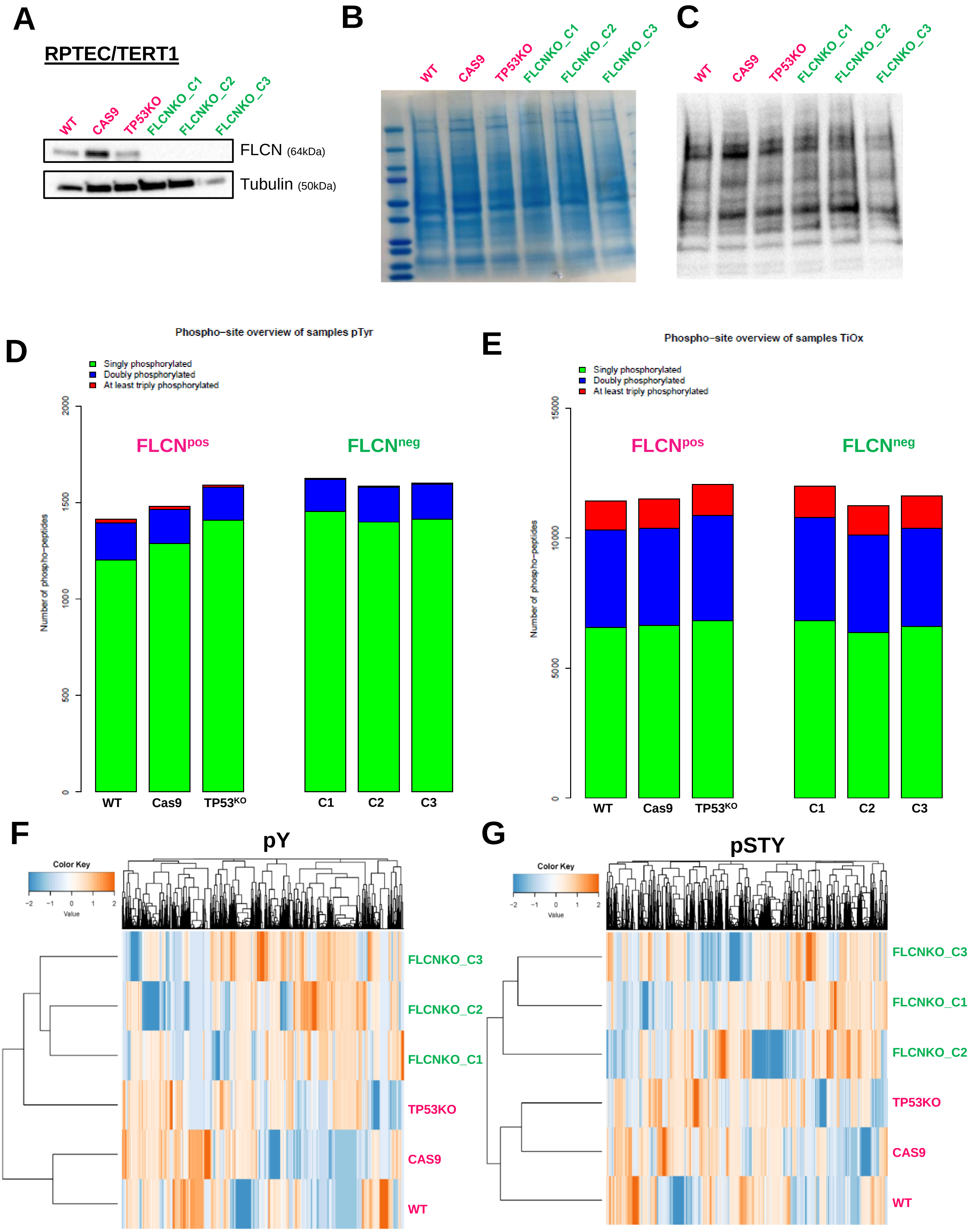

Supplementary figure 1. Comparative phosphoproteomic analyses of RPTEC FLCN^{POS} vs. FLCN^{NEG} cell line pairs.

A) Western blot showing FLCN expression status in six RPTEC/TERT1 cell lines used for phosphoproteomic profiling. Tubulin was used as loading control. **B)** Coomassie staining of gel containing cell line lysates for phosphoproteomic analyses. **C)** Western blot of global tyrosine levels among samples used for phosphoproteomic analyses. **D)** Bar graphs of identified pY phosphosites per individual cell line, with the amount of singly, doubly or triply phosphorylated sites indicated. **E)** Bar graphs of identified pSTY phosphosites per individual cell line, with the amount of singly, doubly or triply phosphorylated sites indicated. **F)** Unsupervised hierarchical cluster analyses of FLCN differential pY peptide intensities. **G)** Unsupervised hierarchical cluster analyses of FLCN differential pSTY peptide intensities.

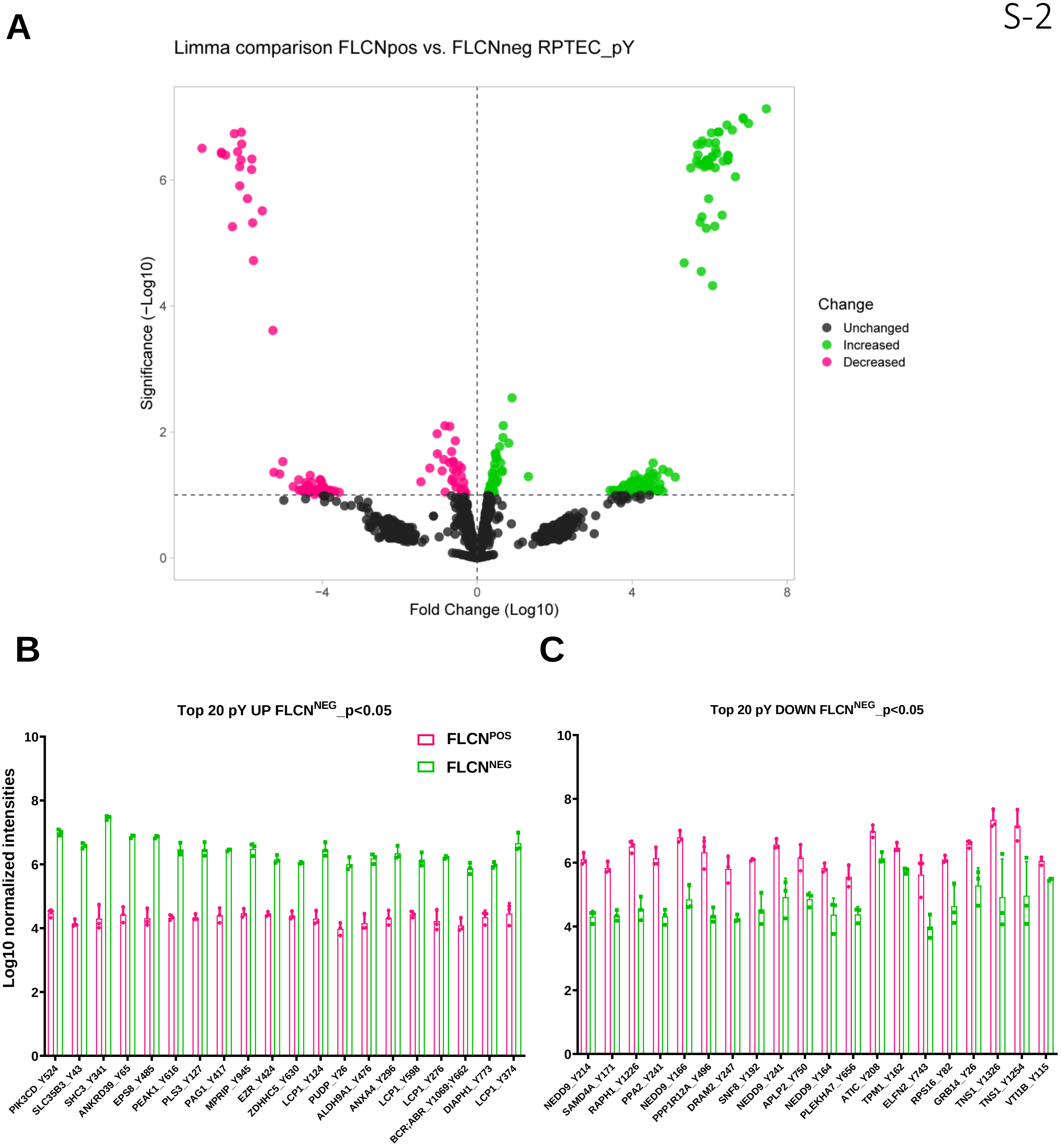

Supplementary figure 2. Volcano plot and top 20 of most significant pY phosphosites that are either higher or lower phosphorylated in FLCN^{NEG} RPTEC.

A) Volcano plot of differential pY phosphosites detected in FLCN^{POS} vs. FLCN^{NEG} comparison. An interactive version of this volcano plot, which is created using the VolcaNoseR [1] web tool, is available via this link.

B) Bar graphs of the top 20 of most significant pY phosphosites that are more phosphorylated in FLCN^{NEG} RPTEC.

C) Bar graphs of the top 20 of most significant pY phosphosites that are less phosphorylated in FLCN^{NEG} RPTEC.

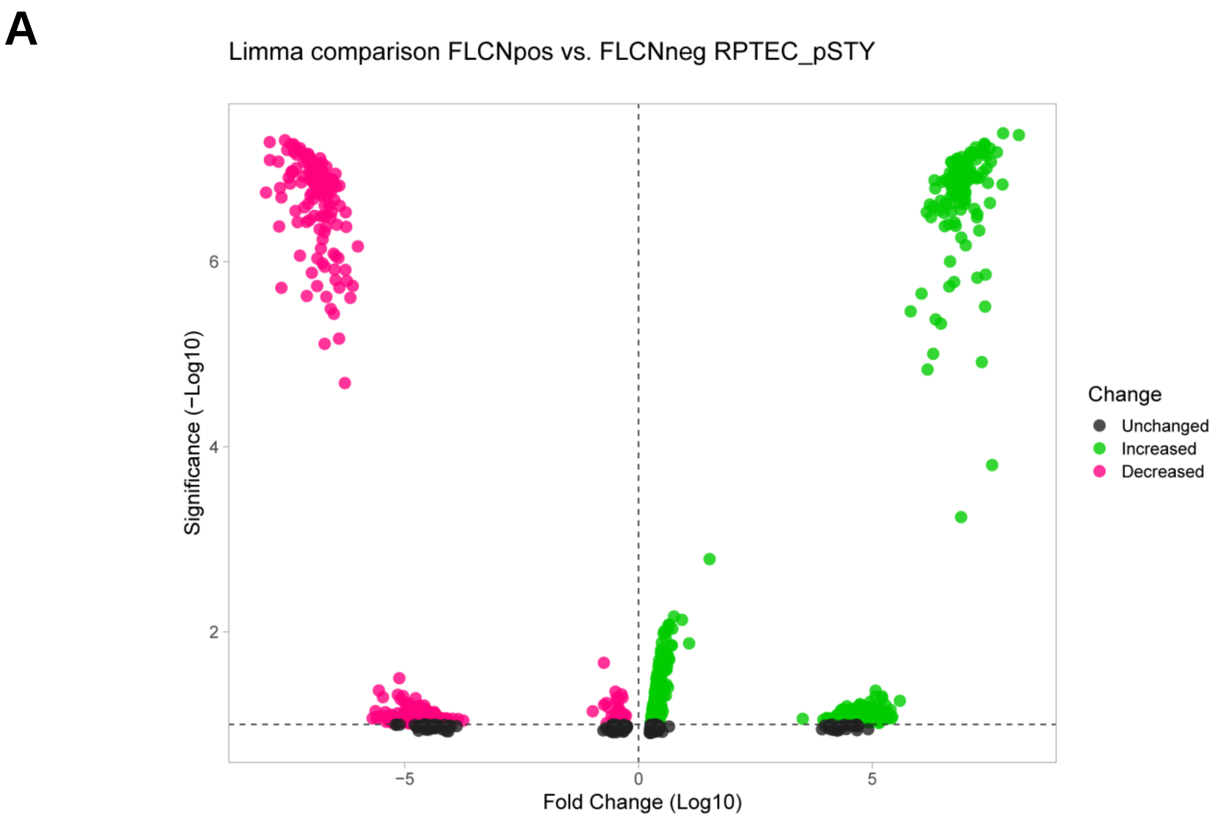

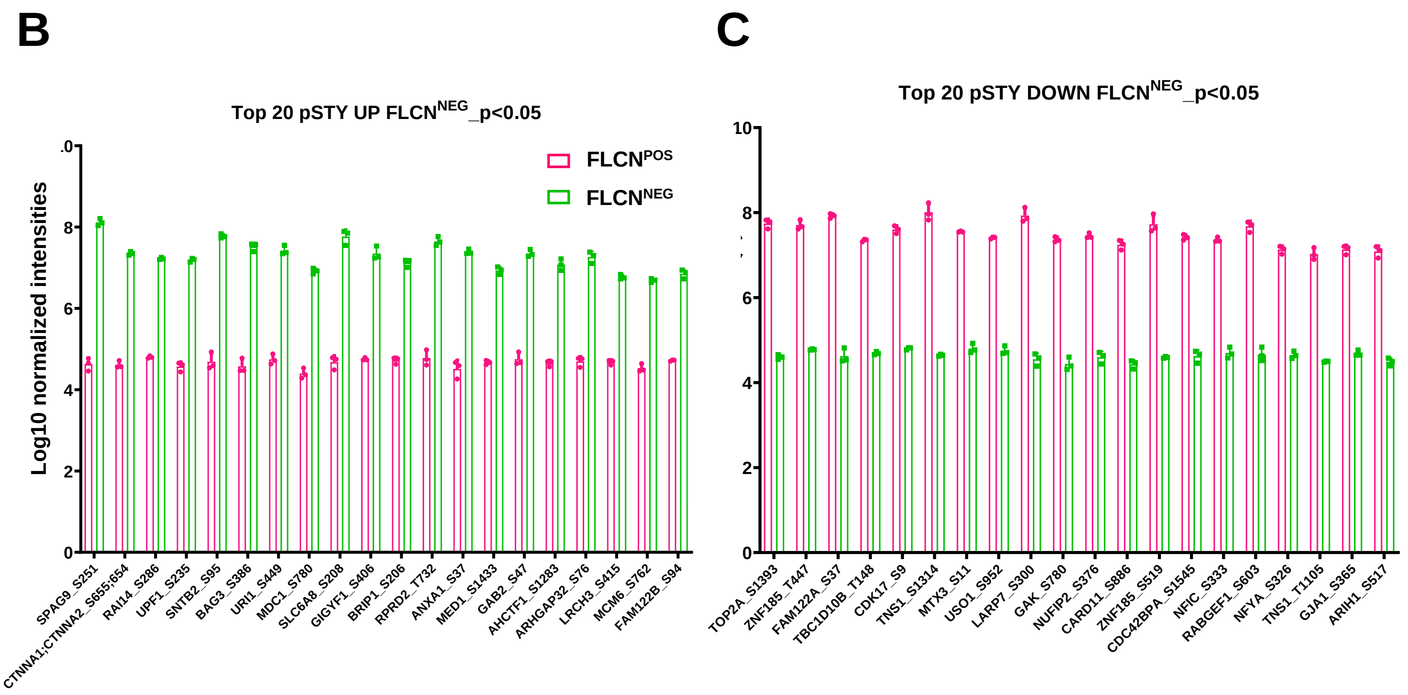

Supplementary figure 3. Volcano plot and top 20 of most significant pSTY phosphosites that are either higher or lower phosphorylated in FLCN^{NEG} RPTEC.

A) Volcano plot of differential pSTY phosphosites detected in FLCN^{POS} vs. FLCN^{NEG} comparison. An interactive version of this volcano plot, which is created using the VolcaNoseR [1] web tool, is available via this link.

B) Bar graphs of the top 20 of most significant pSTY phosphosites that are more phosphorylated in FLCN^{NEG} RPTEC.

C) Bar graphs of the top 20 of most significant pSTY phosphosites that are less phosphorylated in FLCN^{NEG} RPTEC.

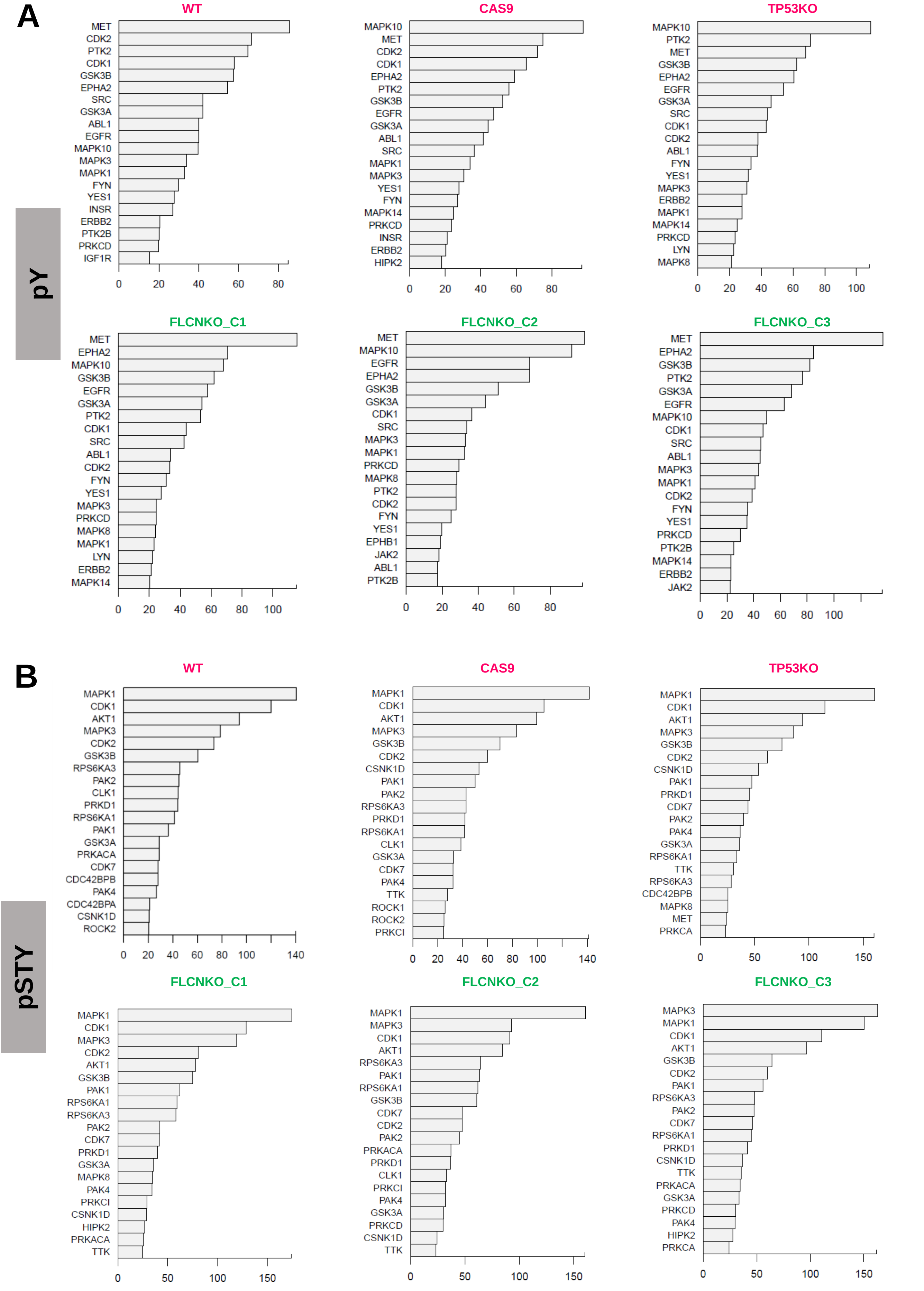

Supplementary figure 4. Top 20 of active kinases identified by INKA per cell line.
A) INKA based ranking of top 20 most active kinases (pY) identified per individual cell line.
B) INKA based ranking of top 20 most active kinases (pSTY) identified per individual cell line.

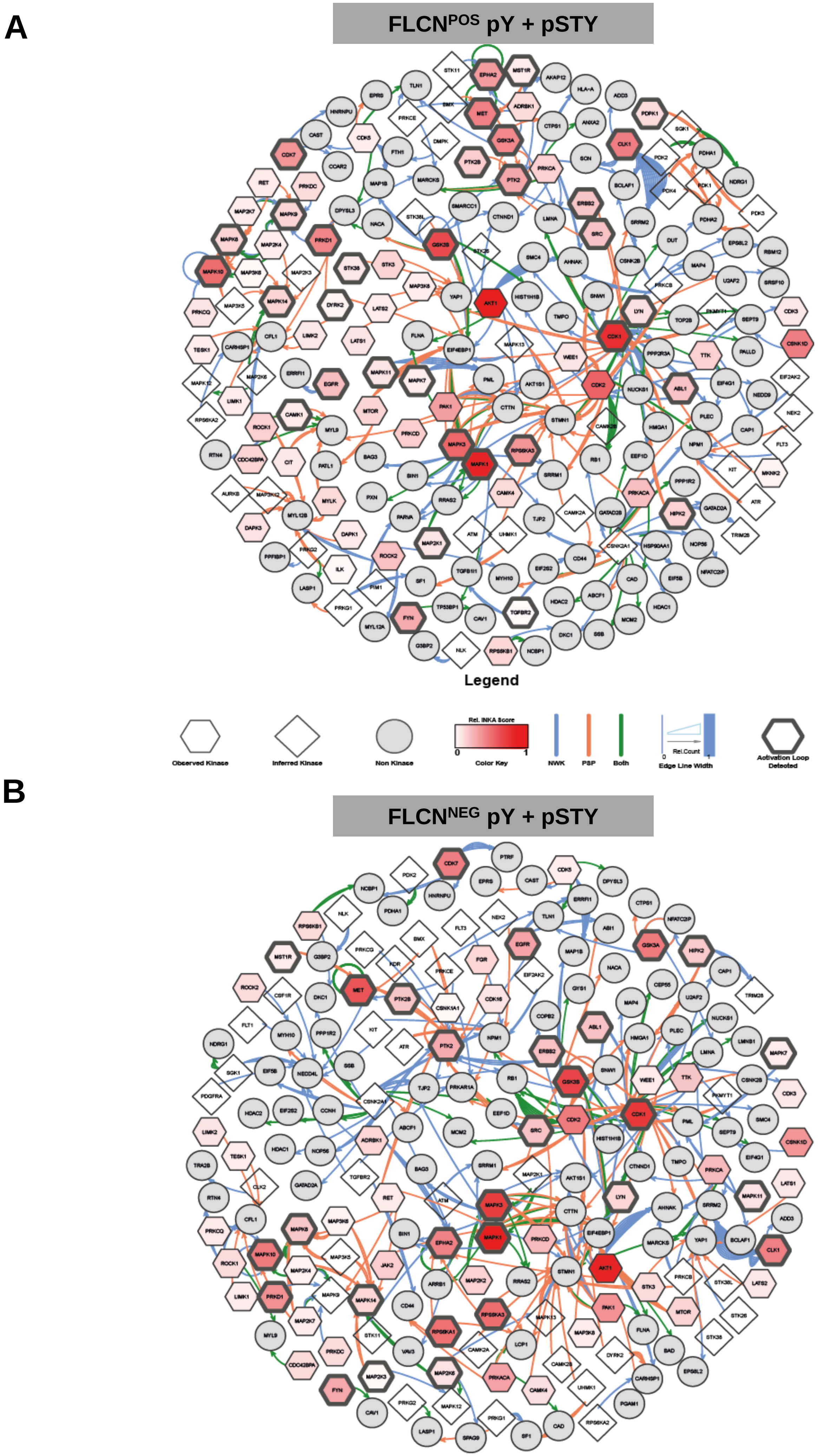
A
FLCN^POS pY + pSTY
Legend
Observed Kinase
Inferred Kinase
Non Kinase
Rel. INKA Score
0
1
Color Key
NWK
PSP
Both
Rel.Count
Edge Line Width
Activation Loop Detected
B
FLCN^NEG pY + pSTY

Supplementary figure 5. Aggregated networks of kinases and substrates identified in FLCN^{POS} and FLCN^{NEG} RPTECs.
A) Aggregated network (pY + pSTY) of kinases and substrates identified in FLCN^{POS}, which are used to calculate aggregated INKA scores (shown in figure 2).
B) Aggregated network (pY + pSTY) of kinases and substrates identified in FLCN^{NEG}, which are used to calculate aggregated INKA scores (shown in figure 2).

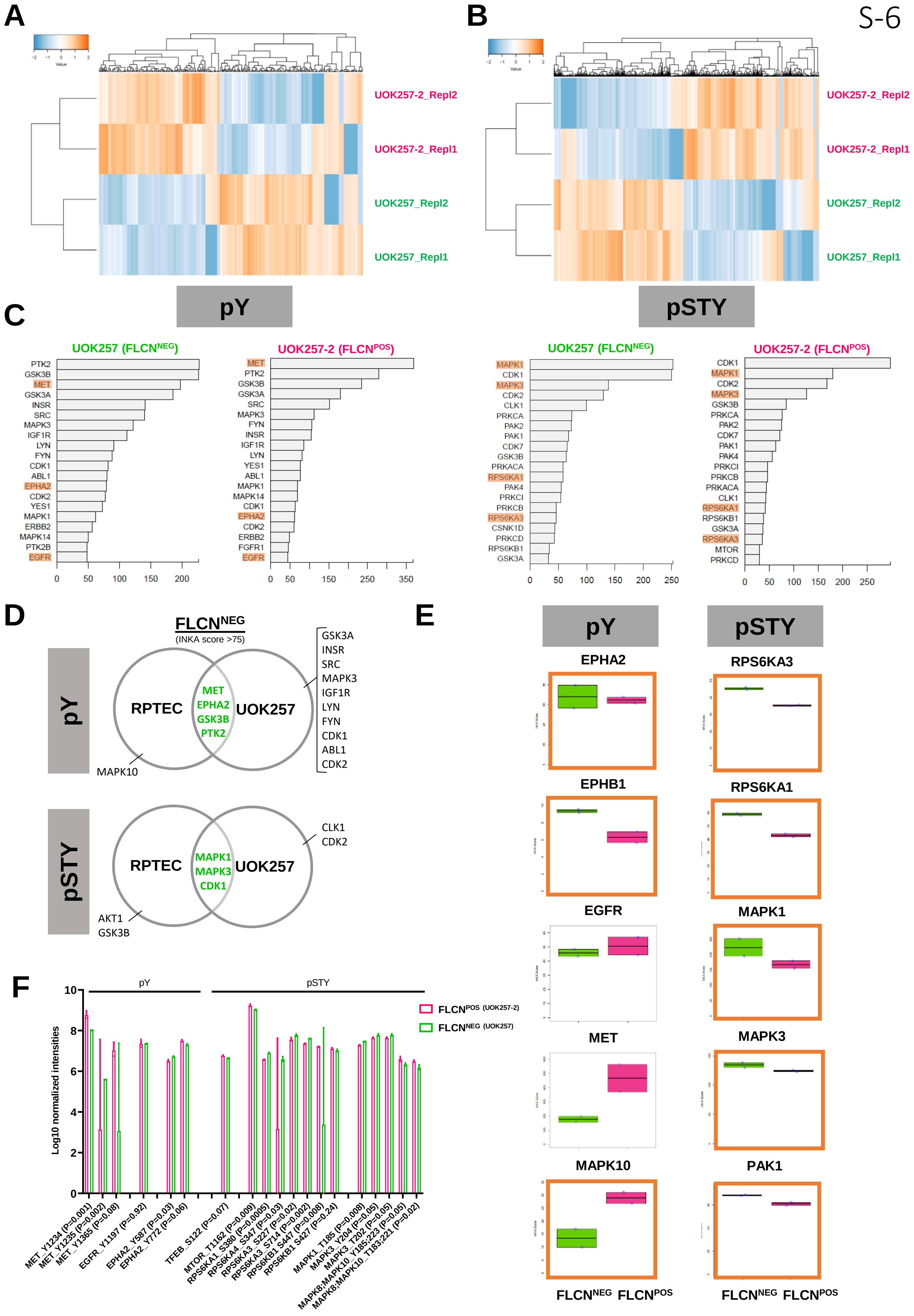
A
B
UOK257-2_Repl2
UOK257-2_Repl1
UOK257_Repl2
UOK257_Repl1
pY
pSTY
C
UOK257 (FLCN^NEG)
UOK257-2 (FLCN^POS)
PTK2 GSK3B MET GSK3A INSR SRC MAPK3 IGF1R LYN FYN CDK1 ABL1 EPHA2 CDK2 YES1 MAPK1 ERBB2 MAPK14 PTK2B EGFR
MET PTK2 GSK3B GSK3A SRC MAPK3 FYN INSR IGF1R LYN YES1 ABL1 MAPK1 MAPK14 CDK1 EPHA2 CDK2 ERBB2 FGFR1 EGFR
MAPK1 CDK1 MAPK3 CDK2 CLK1 PRKCA PAK2 PAK1 CDK7 GSK3B PRKACA RPS6KA1 PAK4 PRKCI PRKCB RPS6KA3 CSNK1D PRKCD RPS6KB1 GSK3A
CDK1 MAPK1 CDK2 MAPK3 GSK3B PRKCA PAK2 CDK7 PAK1 PAK4 PRKCI PRKCB PRKACA CLK1 RPS6KA1 RPS6KB1 GSK3A RPS6KA3 MTOR PRKCD
D
FLCN^NEG
(INKA score >75)
RPTEC
UOK257
MET
EPHA2
GSK3B
PTK2
GSK3A INSR SRC MAPK3 IGF1R LYN FYN CDK1 ABL1 CDK2
MAPK10
MAPK1
MAPK3
CDK1
CLK1
CDK2
AKT1
GSK3B
E
EPHA2
RPS6KA3
EPHB1
RPS6KA1
EGFR
MAPK1
MET
MAPK3
MAPK10
PAK1
FLCN^NEG FLCN^POS
F
pY
pSTY
Log10 normalized intensities
FLCN^POS (UOK257-2)
FLCN^NEG (UOK257)
MET_Y1234 (P=0.001)
MET_Y1235 (P=0.002)
MET_Y1365 (P=0.08)
EGFR_Y1197 (P=0.92)
EPHA2_Y587 (P=0.03)
EPHA2_Y772 (P=0.06)
TFEB_S122 (P=0.07)
MTOR_T1162 (P=0.009)
RPS6KA1_S380 (P=0.0005)
RPS6KA4_S347 (P=0.03)
RPS6KA3_S227 (P=0.02)
RPS6KA3_S714 (P=0.002)
RPS6KB1_S447 (P=0.008)
RPS6KB1_S427 (P=0.24)
MAPK1_T185 (P=0.008)
MAPK3_Y204 (P=0.05)
MAPK3_T202 (P=0.05)
MAPK8;MAPK10_Y185;223 (P=0.05)
MAPK8;MAPK10_T183;221 (P=0.02)

Supplementary figure 6. FLCN-dependent kinase phosphorylation in UOK257 and UOK257-2.

A) Supervised hierarchical cluster analyses of FLCN differential pY peptide intensities. **B)** Supervised hierarchical cluster analyses of FLCN differential pSTY peptide intensities. **C)** INKA based ranking of top 20 most active kinases (pY and pSTY) identified in UOK257 and UOK257-2. Kinases strongly dependent on FLCN expression in RPTEC are highlighted in orange. **D)** Overlap of kinases with highest activity (INKA score >75) in $FLCN^{NEG}$ RPTEC and $FLCN^{NEG}$ UOK257 cells. **E)** Activities of kinases (pY and pSTY) in UOK257 and UOK257-2 that were top differential kinases dependent on FLCN in RPTEC. Activity of EPHA2, EPHB1, RPS6KA3, RPS6KA1, MAPK1, MAPK3, MAPK10 and PAK1 are affected similarly in both cell line backgrounds (boxed orange). EGFR and MET activities appear to be more active $FLCN^{NEG}$ RPTEC but were less active in the $FLCN^{NEG}$ UOK257 tumor cell line. **F)** Normalized intensities of individual FLCN-dependent phosphosites, of which phosphorylation was significantly affected upon FLCN loss in RPTEC, detected in UOK257 and UOK257-2 cell lines too.

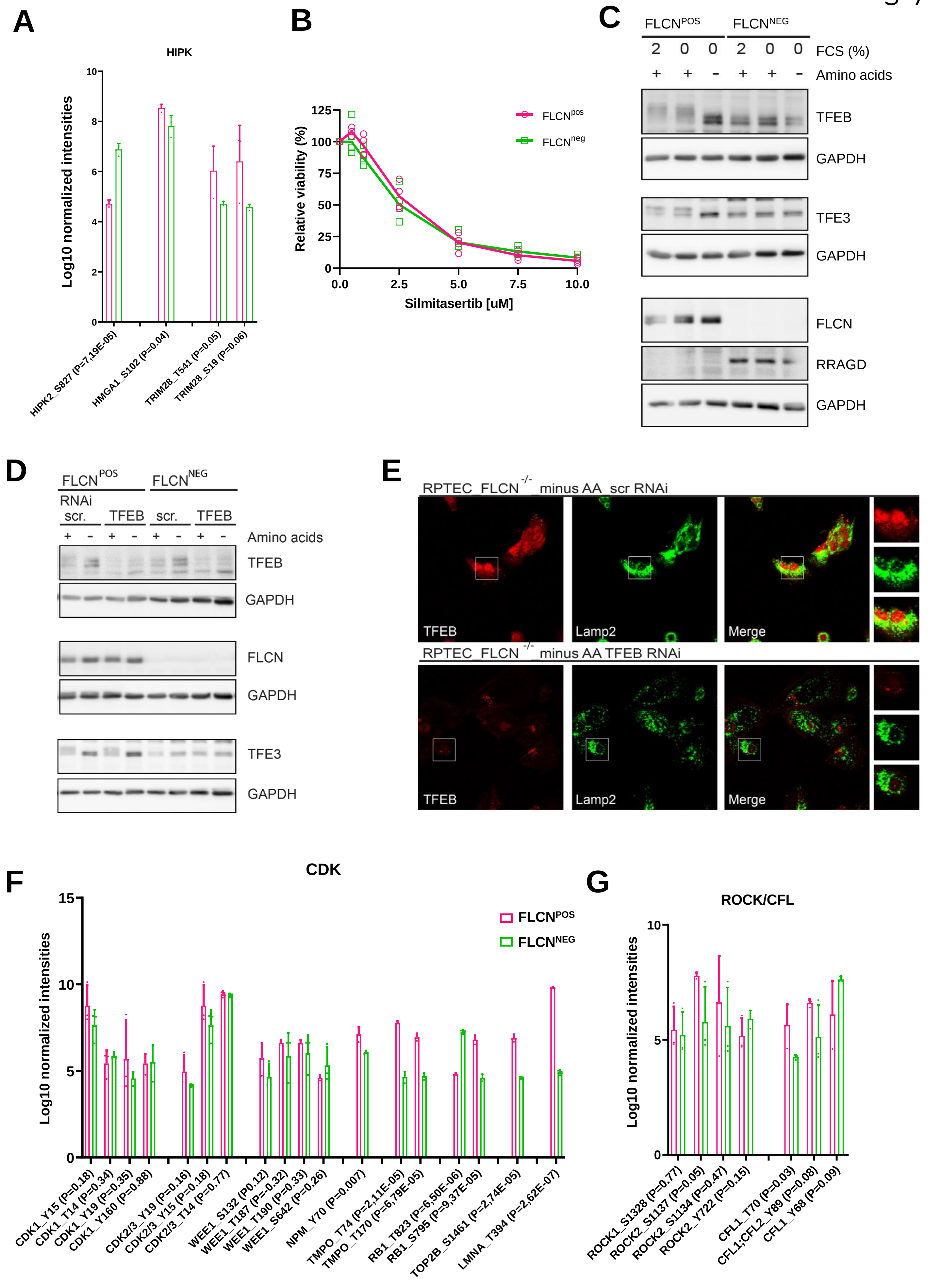
A
HIPK
Log10 normalized intensities
HIPK2_S827 (P=7,19E-05)
HMGA1_S102 (P=0.04)
TRIM28_T541 (P=0.05)
TRIM28_S19 (P=0.06)
B
Relative viability (%)
Silmitasertib [uM]
FLCNpos
FLCNneg
C
FLCNPOS
FLCNNEG
FCS (%)
Amino acids
TFEB
GAPDH
TFE3
FLCN
RRAGD
D
RNAi
scr.
TFEB
Amino acids
E
RPTEC_FLCN-/-_minus AA_scr RNAi
RPTEC_FLCN-/-_minus AA TFEB RNAi
TFEB
Lamp2
Merge
F
CDK
FLCNPOS
FLCNNEG
CDK1_Y15 (P=0.18)
CDK1_T14 (P=0.34)
CDK1_Y19 (P=0.35)
CDK1_Y160 (P=0.88)
CDK2/3_Y19 (P=0.16)
CDK2/3_Y15 (P=0.18)
CDK2/3_T14 (P=0.77)
WEE1_S132 (P0.12)
WEE1_T187 (P=-0.32)
WEE1_T190 (P=0.33)
WEE1_S642 (P=0.26)
NPM_Y70 (P=0.007)
TMPO_T74 (P=2,11E-05)
TMPO_T170 (P=6,79E-05)
RB1_T823 (P=6,50E-06)
RB1_S795 (P=9,37E-05)
TOP2B_S1461 (P=2,74E-05)
LMNA_T394 (P=2,62E-07)
G
ROCK/CFL
ROCK1_S1328 (P=0.77)
ROCK2_S1137 (P=0.05)
ROCK2_S1134 (P=0.47)
ROCK2_Y722 (P=0.15)
CFL1_T70 (P=0.03)
CFL1;CFL2_Y89 (P=0.08)
CFL1_Y68 (P=0.09)

Supplementary figure 7. FLCN dependent HIPK2, CDK1/2 and ROCK1/2 phosphorylation and TFEB/TFE3 western blots.
A) Differential phosphosites of HIPK2 and its substrates HMGA1 and TRIM28. **B)** Silmitasertib dose-response curves of $FLCN^{POS}$ versus $FLCN^{NEG}$ RPTECs show no difference in sensitivity to HIPK2/CK2 inhibition. **C)** Western blot of total TFEB showing a decrease in its phosphorylation levels, indicated by a shift downwards upon amino acid starvation of $FLCN^{POS}$ RPTECs. TFEB is also less phosphorylated in $FLCN^{NEG}$ cells, independent of nutrient availability. TFE3 behaves similarly upon amino acid starvation or FLCN loss in RPTECs. RRAGD expression is upregulated upon FLCN loss and TFEB/TFE3 activation. **D)** siRNA mediated knockdown of TFEB in RPTEC confirms antibody specificity in western blots of TFEB **E)** siRNA mediated knockdown of TFEB in RPTEC $FLCN^{KO}$ confirms antibody specificity in immunofluorescent stainings of TFEB. **F)** Differential phosphosites of CDK1/2 and WEE1 kinases and substrates NPM, TMPO, RB1, TOP2B and LMNA. **G)** Differential phosphosites of ROCK1/2 kinases and CFL1/2
